# Miro2 sulfhydration by CBS/H_2_S promotes human trophoblast invasion and migration via regulating mitochondria dynamics

**DOI:** 10.1038/s41419-024-07167-7

**Published:** 2024-10-26

**Authors:** Hao Feng, Zongxin Sun, Baoshi Han, Huitang Xia, Lumei Chen, Chunlei Tian, Suhua Yan, Yugen Shi, Jie Yin, Wengang Song, Peipei Gong, Shuanglian Wang, Yan Li

**Affiliations:** 1 Department of Obstetrics & Gynecology, the First Affiliated Hospital of Shandong First Medical University & Shandong Provincial Qianfoshan Hospital, Jinan, 250014 China; 2 Department of Emergency, Affiliated Hospital of Chifeng University, Shifeng, 024001 China; 3 Institute of Women, Children and Reproductive Health, Shandong University, Jinan, 250012 China; 4 Jinan Clinical Research Center for Tissue Engineering Skin Regeneration and Wound Repair, Jinan, Shandong 250014 PR China; 5 Department of Cardiology, the First Affiliated Hospital of Shandong First Medical University & Shandong Provincial Qianfoshan Hospital, Jinan, 250014 China; 6 Shandong Provincial Key Laboratory for Rheumatic Disease and Translational Medicine, The First Affiliated Hospital of Shandong First Medical University & Shandong Provincial Qianfoshan Hospital, Jinan, 250012 China; 7 Department of Rehabilitation Medicine, the Provincial Hospital Affiliated to Shandong First Medical University, Jinan, 250021 China; 8 Medical Science and Technology Innovation Center, Shandong First Medical University & Shandong Academy of Medical Sciences, Jinan, 250012 China; 9 Translational Medical Research Centre, the First Affiliated Hospital of Shandong First Medical University & Shandong Provincial Qianfoshan Hospital, Jinan, 250014 China

**Keywords:** Medical research, Physiology

## Abstract

Insufficient cytotrophoblast (CTB) migration and invasion into the maternal myometrium leads to pregnancy related complications like Intra-uterus Growth Restriction (IUGR), and pre-eclampsia (PE). We previously found that hydrogen sulfide (H_2_S) enhanced CTB migration without knowing the mechanism(s) and the pathophysiological significance. By studying human samples and cell line, we found that H_2_S levels were lower in PE patients’ plasma; H_2_S synthetic enzyme cystathionine β-synthetase (CBS) was reduced in PE extravillious invasive trophoblasts. GYY4137 (H_2_S donor, 1 µM) promoted CBS/H_2_S translocation onto mitochondria, preserved mitochondria functions, enhanced cell invasion and migration. CBS knockdown hindered the above functions which were rescued by GYY4137, indicating the vital roles of CBS/H_2_S signal. Disturbance of mitochondria dynamics inhibited cell invasion and migration. The 185 and 504 cysteines of Mitochondrial Rho GTPase 2 (Miro2^C185/C504^) were highly sulfhydrated by H_2_S. Knockdown Miro2 or double mutation of Miro2^C185^/^C504^ to serine fragmented mitochondria, and inhibited cell invasion and migration which can’t be rescued by H_2_S. The present study showed that human cytotrophoblast receives low dose H_2_S regulation; CBS/H_2_S sustained mitochondria functions via Miro2^C185/C504^ sulfhydration to enhance cytotrophoblast mobility. These findings established a new regulatory pathway for cytotrophoblast functions, and provided new targets for IUGR and PE.

## Introduction

Cytotrophoblast (CTB) is one of the most important components in healthy placenta formation. Insufficient CTB migration and invasion into the maternal myometrium delay the transformation of CTB into the spiral artery endothelium which obstructs fetus blood supply, and thus leads to pregnancy related complications like Intra-uterus Growth Restriction (IUGR), pre-eclampsia (PE) and abortion as consequences [1–4]. However, the undefined mechanisms underlying CTB migration and invasion, and the later on transformation of CTB into spiral artery hinders the discovery of efficient therapy strategies to prevent IUGR, PE and abortion in clinics.

We have previously found the gaseous transmitter, hydrogen sulfide (H_2_S), rescued fetus early-stage abortion in CBS^+/−^ mice, as well as enhanced CTB (HTR-8/SVneo cell line) migration in vitro [5]. But the detailed mechanism(s) and the pathophysiological significance of H_2_S on CTB migration are totally unknown. H_2_S in serum from PE patients was reported to be significantly decreased [6] compared with that from normal pregnancy indicating the clinical relevance of H_2_S in PE. Meanwhile, placenta samples from PE patients demonstrated shallow CTB migration and invasion [7] into the maternal myometrium as well as morphological disorders in mitochondria of CTB. Whether the decreased H_2_S leads to shallow CTB migration and mitochondria disorder in placenta needs further investigation.

Intracellularly, H_2_S is synthesized by three enzymes with tissue and cell specific pattern. These enzymes are, cystathionine γ-lyase (CSE), which mainly functions in the cardiovascular system, cystathionine β-synthetase (CBS) in the central nervous system and 3-mercaptopyruvate sulfurtransferase (MST3) in mitochondria in mammals. However, human plasma H_2_S comes from not only tissues, but from gut microbiome [8, 9]. Although the published data has indicated that the decreased CBS expression lowered H_2_S production in PE patients [10], we would still question if the decreased circulating H_2_S is a result or cause of the reduced CBS in the placenta. We have demonstrated the low concentration of H_2_S (less than 5 µM) stimulated CBS aggregation in trophoblasts indicating low H_2_S maintains CBS protein level as well as tissue H_2_S production.

CSE/CBS can be translocated onto mitochondria and protect smooth muscle cells via CSE/H_2_S-ATP synthesis [11] or CBS/H_2_S antioxidation [12]. However, in hypoxia, prolonged CBS/H_2_S translocation onto mitochondria lead to mitochondria swelling and inhibited Ca^2+^-induced cyt c release in human hepatoma Hep3B cells [13]. These data implicate CSE/CBS-H_2_S signal determines cell fates based on cell type and stress state. Our study showed the CBS/H_2_S signal preserved mitochondria functions and enhanced cell invasion and migration of human trophoblasts in unstressed state.

With the question whether CBS/H_2_S signal promoted CTB invasion and migration via regulating mitochondria functions, we designed this study to explore: 1) if CBS/H_2_S signal promotes CTB invasion and migration; 2) whether CBS/H_2_S signal regulates mitochondria function and the detailed mechanisms.

## Results

### Decreased cystathionine β-synthetase (CBS)/H2S production in cytotrophoblast and plasma of PE patients

**Fig. 1:**
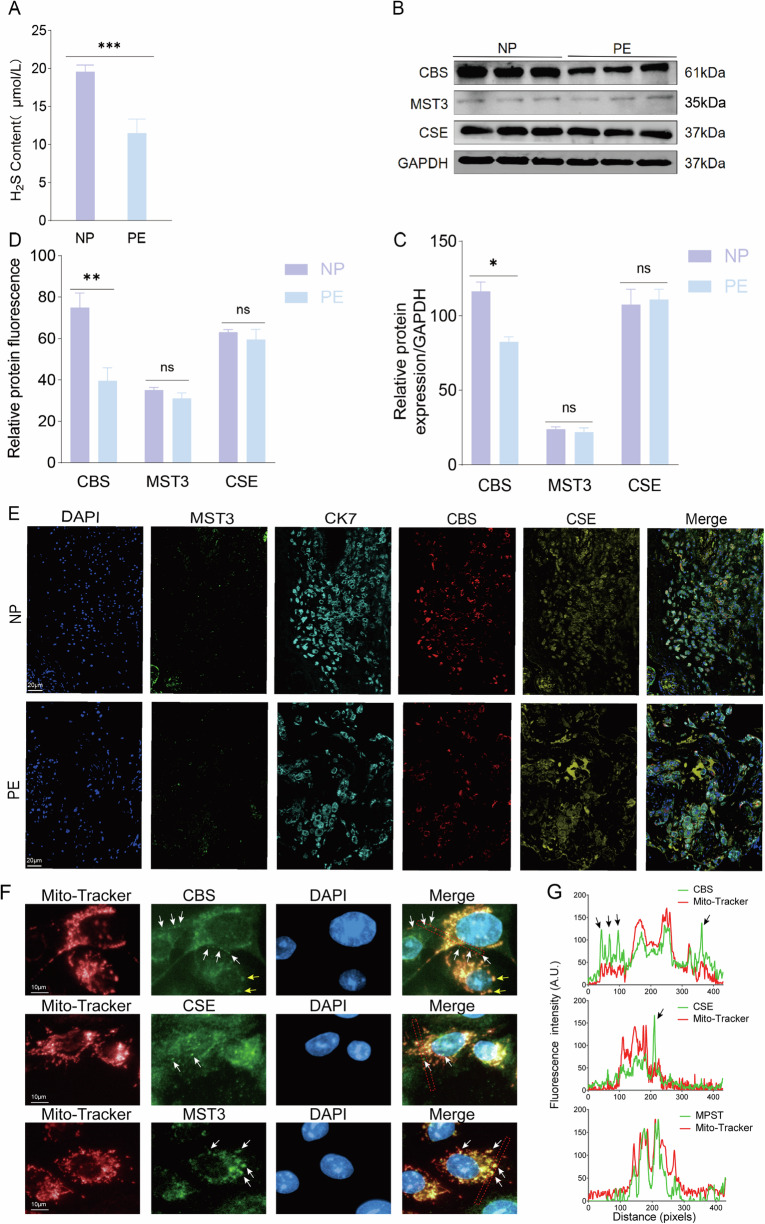
Plasma H_2_S and H_2_S synthetic enzymes expression in human placenta and trophoblast cell line. **A** Maternal plasma H_2_S content was detected in preeclampsia (PE) patients (*n* = 10) and normal pregnancy (NP) women (*n* = 10). **B**, **C** CBS, CSE and MST3 proteins were detected from human placentas extracts using western blot. The relative expression of the 3 proteins were statistically analyzed in the bar graph. PE placenta: *n* = 6; NP placenta: *n* = 6. **D**, **E** Representative immunofluorescent images of triple staining of CBS (red), CSE (yellow) or MST3 (green) with CK7 (light blue) and DAPI (dark blue, nuclei) in human placenta. Panel **D** showed the statistical analysis of fluorescent intensity indicating CBS, CSE and MST3 colocalized with CK7 in **E**. The CBS fluorescent intensity in CK7 positive area was significantly decreased in placenta from PE patient. PE placenta: *n* = 6; NP placenta: *n* = 6. **F** Immunofluorescent staining with Mito-Tracker (red) and CBS/CSE/MST3 (green) in HTR-8 cells. White arrow showed the positive staining of CBS and CSE. Yellow arrow showed double labeling of CBS and Mito-Tracker. **G** Colocalization analysis of Mito-Tracker and CBS/CSE/MST3 in the red dashed square of **F**. Black arrow showed positive staining of CBS/CSE. ***P < 0.001; **P < 0.01; *P < 0.05.

To determine the circulating H_2_S level and H_2_S synthetic enzyme expression in human placentas, we measured H_2_S level with MMBS methods and tested the protein levels and cellular distribution of CBS, CSE, and MST3 in placentas from PE and normal pregnancy women. The results showed a significant decrease (~50% reduction) of plasma H_2_S from PE patients comparing with that from normal pregnancy women (Fig. 1A). Meanwhile, CBS, CSE, and MST3 were all detected in human placenta, but only CBS expression was significantly decreased in the PE placenta compared with the placenta of normal pregnancy women (NP) (Fig. 1B, C). Then, we assessed the distribution of CBS, CSE and MST3 in placenta. The immunofluorescence results showed CBS and CSE were co-localized with CK-7 marked cells (extravillous invasive trophoblast, EVT marker) in placenta, while MST3 were less in CK7 positive cells (Fig. 1D, E). EVTs were mainly involved in spiral artery remodeling, thus we tested if there was different expression of CBS, CSE and MST3 in EVTs from both NP and PE placentas. Double staining with invasive cytotrophoblast marker CK7 and CBS/CSE/MST3 antibodies showed that CBS was decreased largely in EVTs from PE placenta (Fig. 1D, E) indicating there is disturbance of CBS expression in EVTs of PE placenta. We would question if the reduced circulating H_2_S is a result or cause of reduced CBS expression in PE patients.

**Fig. 2:**
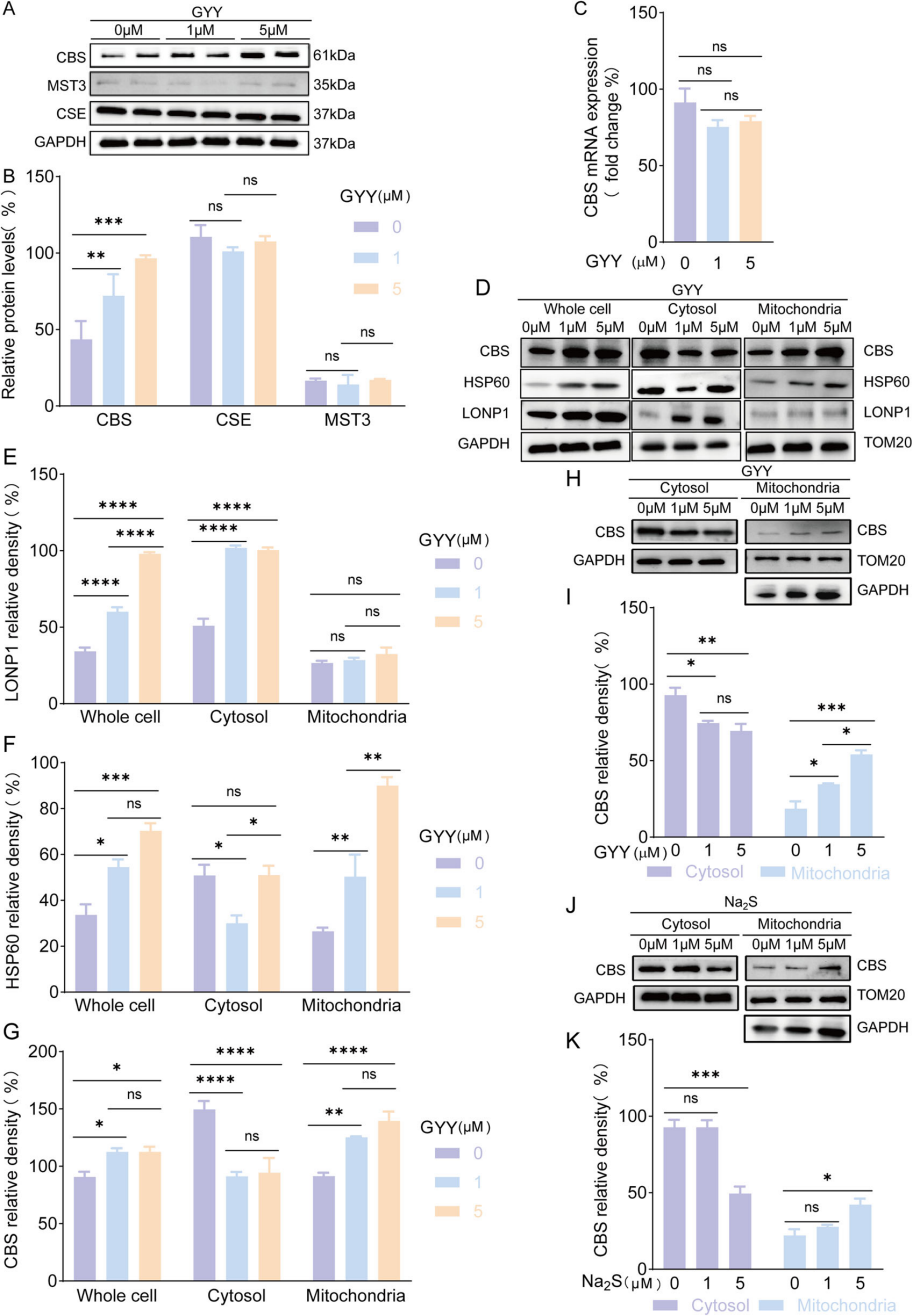
H_2_S enhanced CBS expression and distribution on mitochondria and reduced CBS degradation. **A**, **B** Representative western blot bands of anti-CBS, anti-CSE and anti-MST3 blotting in HTR-8/SVneo trophoblast extracts. Statistical bar graph showed the relative protein expression of CBS, CSE and MST3. GYY4137 at 1 and 5 µM enhanced CBS expression in HTR-8/SVneo. *n* = 7 each group. **C** mRNA level of CBS was evaluated in low dose GYY4137 (1 µM, 5 µM) treatment. *n* = 3 for each group. **D**–**G** Western blot method detected distribution and expression levels of CBS, LONP1 and HSP60 in HTR-8/SVneo. Bar graph showed the differential expression of CBS, LONP1 and HSP60 in whole cell, cytosol and mitochondria in response to low dose GYY4137 (1 µM, 5 µM) stimuli separately. *n* = 6 each group. **H**–**K**, CBS expression was detected in cytosol and mitochondria separately with or without GYY4137/Na_2_S (1 µM, 5 µM) treatment in HTR-8/SVneo. Bar graph showed decreased distribution of CBS in cytosol and increased in mitochondria. CBS expression was downregulated in cytosol and increased in mitochondria at 5 µM Na_2_S. *n* = 7 each group. ‘ns’: no significance. *P < 0.05; **P < 0.01; ***P < 0.001; ****P < 0.0001.

To evaluate the significance and bioactivity of the CBS/H_2_S signal in cytotrophoblast, we addressed the cytotrophoblast cell line, HTR-8/SVneo, as our in vitro cell model for further investigation. As predicted, HTR-8/SVneo expressed CBS, CSE and MST3, and the three enzymes located in the cytosol and mitochondria by double labeling the cells with antibodies to the three enzymes and mitochondria marker (Mito-Tracker) (Fig. 1F). Colocalization analysis showed CBS and CSE locates both in mitochondria and cytosol. (Fig. 1G). Interestingly, we found that low dose of exogenous H_2_S donor application (GYY4137, 1 and 5 µM) enhanced CBS expression without altering CSE and MST3 levels (Fig. 2A, B), implying CBS protein level can be regulated by low dose H_2_S. This finding helped us to clarify the cause-and-effect theory partially because of the reduced circulating H_2_S led to down-regulate of tissue CBS expression although the original risk factor is a mystery for now.

To find out how H_2_S leads to increased CBS expression, we evaluated the transcriptional expression of the gene *CBS*. However, CBS mRNA didn’t alter obviously in response to GYY4137 (Fig. 2C) indicating there was an alternative pathway regulating CBS protein accumulation or degradation instead of via CBS transcription. It has been reported mitochondria CBS degradation was hindered by Lon protease 1 (LONP1) [13]. We tested LONP1 expression in HTR-8/SVneo. The results showed LONP1 was not changed in response to GYY4137. However, heat shock protein 60 (HSP60) was tested and increased significantly after GYY4137 application (Fig. 2D–G). HSP60 was reported to inhibit LONP1 activity in mitochondria. This data suggested that the enhanced CBS aggregation by low H_2_S was mediated by upregulated HSP60 in HTR-8/SVneo. Then we assessed the CBS distribution in the cellular compartment to find out if CBS localized differently in the cell. Interestingly, the results showed CBS predominantly aggregated with mitochondria (Fig. 2H, I). While GYY4137 decreased CBS distribution in the cytosol without mitochondria (Fig. 2D, G, H, I), and didn’t alter CBS expression in nuclei (Fig. S1A). The other H_2_S donor, Na_2_S, was addressed to confirm the CBS response to H_2_S donors. The CBS protein was increased in mitochondria after Na_2_S incubation (Fig. 2J, K) which confirmed that H_2_S donors enhanced CBS expression in HTR-8/SVneo mitochondria.

### CBS/H_2_S signal on mitochondria morphology and functional regulation

**Fig. 3:**
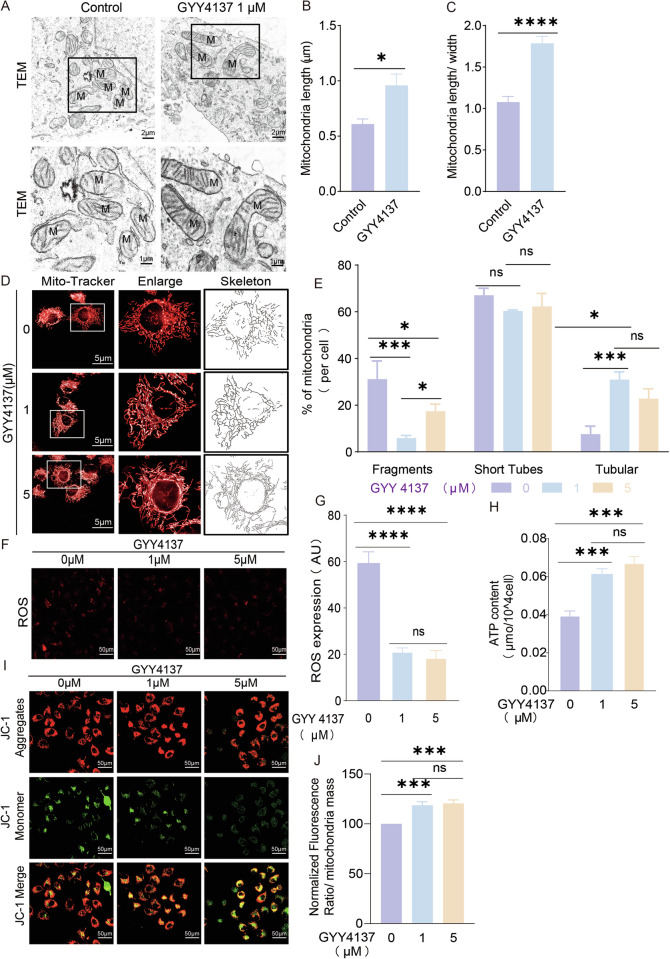
H_2_S donor regulated mitochondria morphology and improved mitochondria functions. **A** Representative TEM images of mitochondria morphology with and without GYY4137 (1 µM) treatment. Mitochondria size was small and even in Control group, and mitochondria were elongated and cristae was clear and long in GYY4137 group. ‘M’: mitochondria. **B**, **C** Statistical bar graph of **A**, mitochondria length and length/ratio were significantly increased in GYY4137 group. *n* = 6 each group. **D** Representative images of live-cell mitochondria tracking with low dose GYY4137 (1 µM and 5 µM) using confocal microscope. The mitochondria tubular was elongated in GYY4137 groups. **E** Statistical bar graph of mitochondria morphology changes. Fragments were decreased and tubular increased in GYY4137 groups. *n* = 10 each group. **F**, **G** ROS production detection with fluorescent detector (red). Fluorescent density was significantly decreased after GYY4137 application. **H** ATP content was increased significantly in GYY4137 groups. *n* = 7 each group. **I**, **J** Mitochondria membrane potential (Δψm) was indicated with fluorescent detector JC-1. Red fluorescence was detected aggregated more on mitochondria in GYY4137 groups. *n* = 7 each group. *P < 0.05; ***P < 0.001; ****P < 0.0001.

To investigate the functional significance of CBS translocation to mitochondria, we analyzed mitochondrial morphological alterations followed by stimulation with low concentration of H_2_S donor GYY4137 (1 µM and 5 µM). A transmission electron microscope (TEM) study showed mitochondria length/width ratio and length increased significantly after GYY4137 treatment (1 µM) (Fig. 3A–C). Mitochondria cristae were clear and cristae numbers were increased in GYY4137 treated cells (Fig. 3A, lower panel). The total mitochondria numbers didn’t change much in control and GYY4137 treated cells (Fig. S1B). Live-cell mitochondria tracker analysis also revealed a significant decrease in fragmentation and increase of tubular formation after treatment with GYY4137 (Fig. 3D, E). Following the morphological changes, ROS production was decreased (Fig. 3F, G) and the ATP level was increased significantly in the GYY4137 treated group compared with that in control group (Fig. 3H), indicating CBS-mitochondria translocation protected cells from energy deprivation and ROS injury which were the general cell injury compartments. Meanwhile, low dose GYY4137 maintained the mitochondria membrane potential (Δψm) at a relatively high level (Fig. 3I, J), indicating low H_2_S preserved energy production and mitochondria stability in HTR-8/SVneo.

**Fig. 4:**
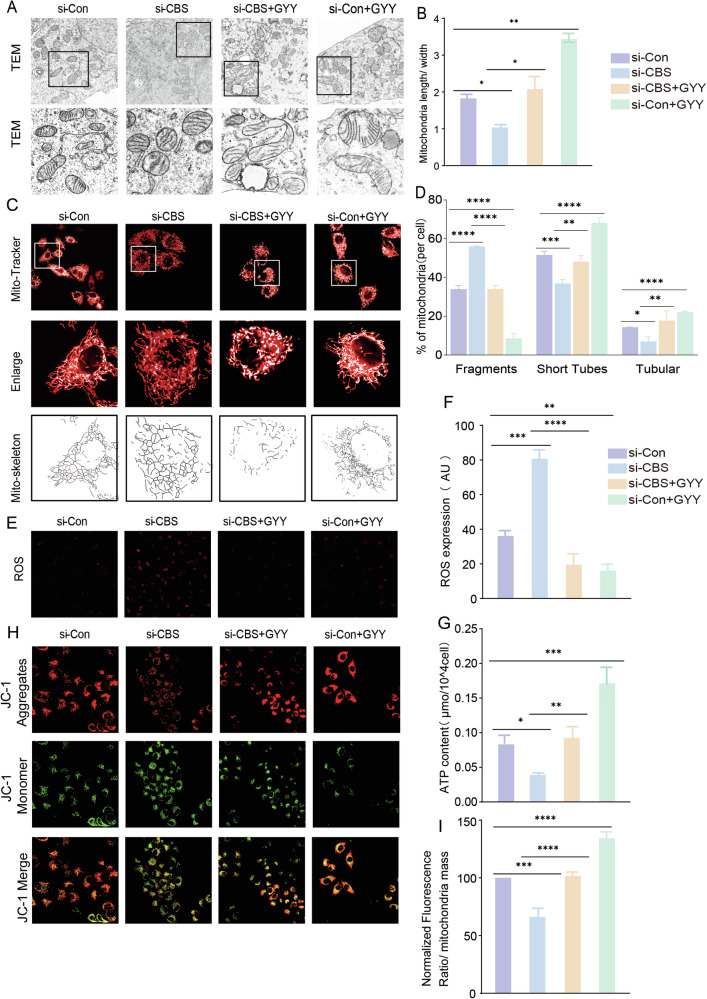
CBS/H_2_S on mitochondria morphology and mitochondria functions. **A**, **B** TEM image represented mitochondria size and shape were even in si-Control group. But mitochondria size and shape were unregular and not even in si-CBS. Mitochondria were elongated in si-CBS + GYY4137 group. Bar graph showed the statistical analysis of mitochondria length/width between groups. **C**, **D** Mitochondria tubular and short tubes were down regulated, while fragmentation was increased in si-CBS group. *n* = 7 each group. **E**, **F** ROS production detection after CBS knockdown by si-CBS. ROS level was elevated in si-CBS group. ROS production was decreased in si-CBS + GYY4137. *n* = 7 each group. **G** ATP production was reduced in si-CBS group but increased in si-CBS + GYY4137 group. *n* = 7 each group. **H**, **I** Δψm was decreased in si-CBS group, but increased in si-CBS + GYY4137 group. *n* = 7 each group. *P < 0.05; **P < 0.01; ***P < 0.001; ****P < 0.0001.

To study the functional significance of CBS-translocation onto mitochondria and if CBS exerts a protective function via H_2_S production, we used small interfering RNA targeting CBS to evaluate the morphological changes, ATP and ROS production, Δψm as well as H_2_S production in HTR-8/SVneo cells. We designed and selected the most efficient si-CBS to knockdown the *CBS* translation (Fig. S1C). The mitochondria exhibited less cristae, tubular and more fragments in the si-CBS knockdown group compared with si-Control group (Fig. 4A–D), while mitochondria in si-CBS + GYY4137 group had more tubulars and less fragments than si-CBS group (Fig. 4C, D) indicating GYY4137 rescued si-CBS induced mitochondria morphological changes. TEM data showed a significantly decreased mitochondria length/width ratio in the si-CBS group compared with that in the si-Control group; GYY4137 increased the mitochondria length/width ratio in the si-CBS group (Fig. 4A, B). Mitochondria numbers didn’t change much among si-Control, si-CBS and siCBS +GYY4137 groups (Fig. S1D).

Mitochondria functional study showed the ROS production was increased in si-CBS group compared with that in the si-Control group (Fig. 4E, F). Also, ATP production and Δψm were significantly decreased in the si-CBS group compared with that in si-Control group (Fig. 4G–I). While GYY4137 inhibited ROS production, enhanced ATP production and preserved Δψm in si-CBS + GYY4137 group comparing with si-CBS group (Fig. 4E–I) suggesting CBS protected mitochondria functions via H_2_S.

H_2_S probe assay showed that H_2_S production was decreased in si-CBS transfected cells comparing with that from si-Control group (Fig. S1G). Seahorse experiment was addressed to detect mitochondria oxygen consumption rate. The basal, ATP linked, and maximal respirations of HTR-8/SVneo were significantly decreased in si-CBS group compared with si-Control group, while basal, ATP linked, and maximal respirations were increased in si-CBS + GYY4137 group compared with si-CBS group with statistical significance (Fig. S2C–F). The decreased H_2_S production, and morphological and functional recovery of mitochondria by H_2_S donor indicated CBS/H_2_S signal contributed largely to cell function via regulating mitochondria morphology and functions.

### CBS/H_2_S signal enhanced HTR-8/SVneo invasion and migration via regulating mitochondria functions

**Fig. 5:**
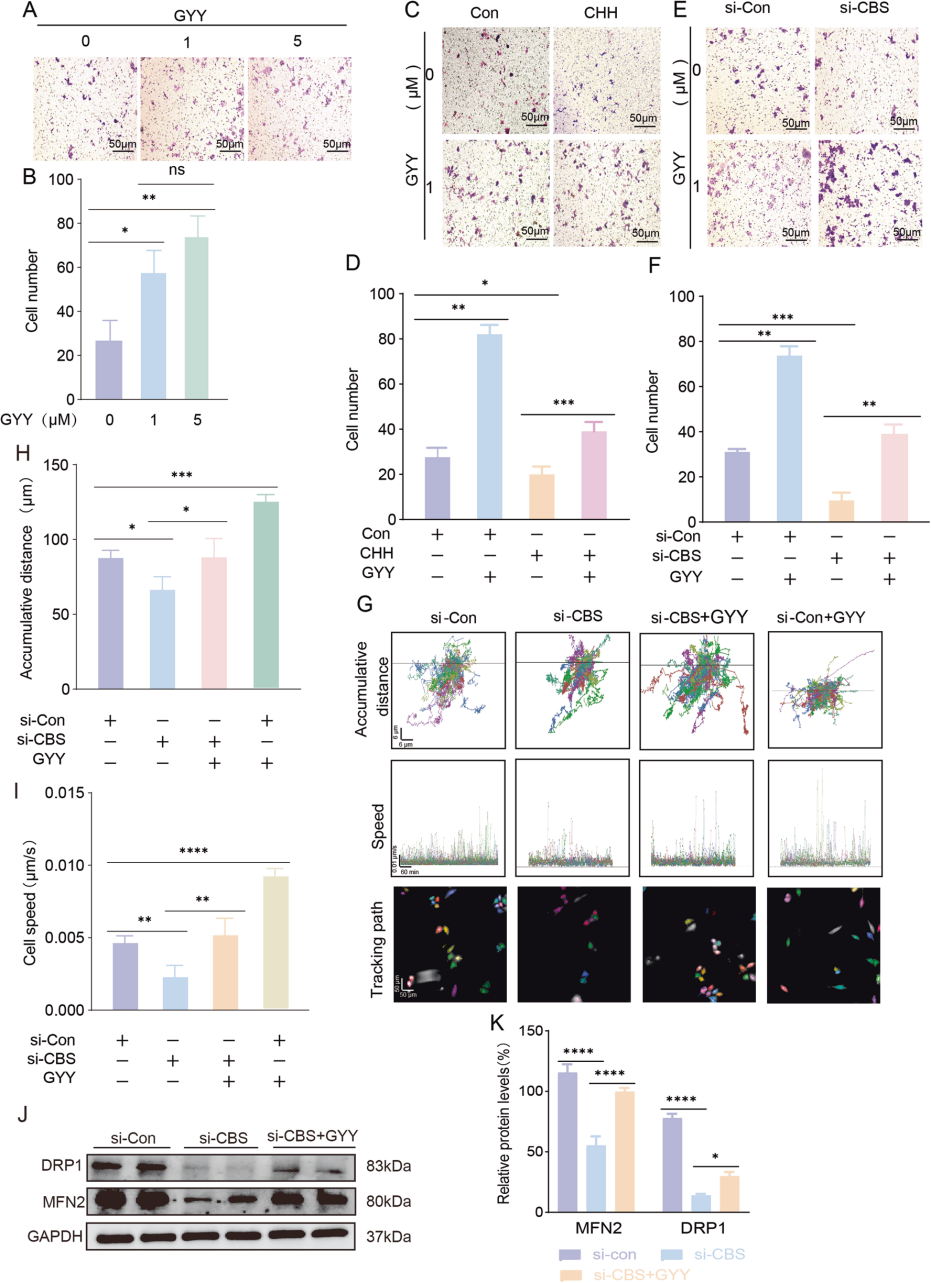
CBS/H_2_S enhanced HTR-8/SVneo trophoblast invasion and migration. **A**, **B** Transwell study of HTR-8/SVneo presented the penetrated cells stained with crystal violet. Penetrated cell numbers were increased significantly in GYY4137 group. *n* = 6 each group. **C**, **D** CBS selective blocker CHH on HTR-8/SVneo invasion. CHH inhibited cell penetration without stimuli. GYY4137 at 1 µM increased cell penetration in the presence of CHH. *n* = 6 each group. **E**, **F** Cell penetration through transwell was decreased in si-CBS group, and increased in si-CBS + GYY4137. *n* = 6 each group. **G**–**I** Live-cell tracking distance and speed were recorded and analyzed with confocal microscope. The accumulative distance demonstrating the total distance that cells moved in about 16 hours. The speed demonstrated the mean moving speed of cells. Each dot indicated one analyzing point of the current speed. Tracking path showed the tracked cells and the moving path. The accumulative distance and moving speed were decreased in si-CBS groups, and increased in si-CBS + GYY4137 group. At least 6 fields per well were analyzed and *n* = 6 each group. **J**, **K** MFN2 and Drp1 expression in response to si-Con, si-CBS and si-CBS + GYY. *n* = 3 each group. *P < 0.05; **P < 0.01; ***P < 0.001; ****P < 0.0001.

We have previously reported that H_2_S donor, GYY4137, enhanced HTR-8/SVneo invasion [5] in vitro without knowing the exact mechanisms. Transwell study was used to analyze HTR-8/SVneo invasion. Live cell tracking was used to assess HTR-8/SVneo migration accumulative moving distance and speed under a confocal microscope. In consistence with our previous report, lower concentration of GYY4137 (1 µM and 5 µM) promoted HTR-8/SVneo invasion (Fig. 5A, B). Both CBS antagonist CHH (Fig. 5C, D) and si-CBS transfection (Fig. 5E, F) hindered HTR-8/SVneo invasion while GYY4137 recovered CHH and si-CBS induced reduction of cell invasion (Fig. 5C–F). The HTR-8/SVneo moving distance was recorded and analyzed for about 16 hours. The cell moving path was dotted with Harmony 4.9 software. The accumulative distance and moving speed of the tracked cells were significantly decreased in the si-CBS group, while GYY4137 rescued si-CBS induced decreases in accumulative distance and moving speed (Fig. 5G–I). These data indicated that CBS/H_2_S enhanced HTR-8/SVneo invasion and migration.

To confirm if CBS/H_2_S promoted HTR-8/SVneo invasion and migration via regulating mitochondria functions, we investigated the roles of mitochondria in cell invasion and migration. Mitochondrial division inhibitor 1 (Mdivi-1) [14] to inhibit mitochondria fission, and Mitofusion 2 siRNA to disrupt mitochondria fusion were used to study mitochondria activity on cell invasion and migration. By evaluating proteins that are involved in mitochondria dynamics, we found that mitofusion 1 and 2 (MFN1 and MFN2, mediating mitochondria fusion), and dynamin-related protein 1 (Drp1, mediating mitochondria fission) were detected in HTR-8/SVneo (Fig. S3A). But MFN2 was detected to increase after GYY4137 treatment, while Drp1 was decreased in response to GYY4137 (Fig. S3A, B) indicating H_2_S also regulates mitochondria dynamics. Blocking Drp1 activity with Mdivi-1 increased cell invasion significantly (Fig. S3C, D), and cell migration distance and speed were also increased in Mdivi-1 treated group compared with those in the control group (Fig. S3E–G). Again, we used MFN2 siRNA (Fig. S3H), to interfere mitochondria fusion in which mitochondria fusion enhanced mitochondria function and improved cell activity as a consequence. The results showed that MFN2 siRNA inhibited HTR-8/SVneo invasion (Fig. S3I, J) and reduced cell migration distance and speed (Fig. S3K–M). Knocking down CBS led to decreased MFN2 and increased Drp1 expression in HTR-8/SVneo cells (Fig. 5J, K) suggesting CBS modulates mitochondria dynamics via MFN2 and Drp1. To explore how CBS/H_2_S regulated MFN2 and Drp1 protein, qRCR was conducted to assess the transcriptional regulation of the proteins, and protein stability was analyzed as well. The data showed a decreased MFN2 mRNA and increased Drp1 in si-CBS, while GYY4137 rescued the decreased MFN2 and increased Drp1 mRNA (Fig. S3O). While the MFN2 and Drp1 degradation was not altered in Control and GYY4137 treated group (Fig. S3P–R). Thus far, these data demonstrated that mitochondria dynamics of fusion and fission played key roles in CBS mediated HTR-8/SVneo invasion and migration.

Combining with the data that si-CBS destroyed mitochondria tubular and increased fragmentation, while H_2_S donor GYY4137 rescued morphological and functional changes of mitochondria, we concluded that the CBS/H_2_S signal enhanced HTR-8/SVneo invasion and migration via regulating mitochondria dynamics.

### Miro-2 sulfhydration on CBS/H_2_S mediated mitochondria regulation

**Fig. 6:**
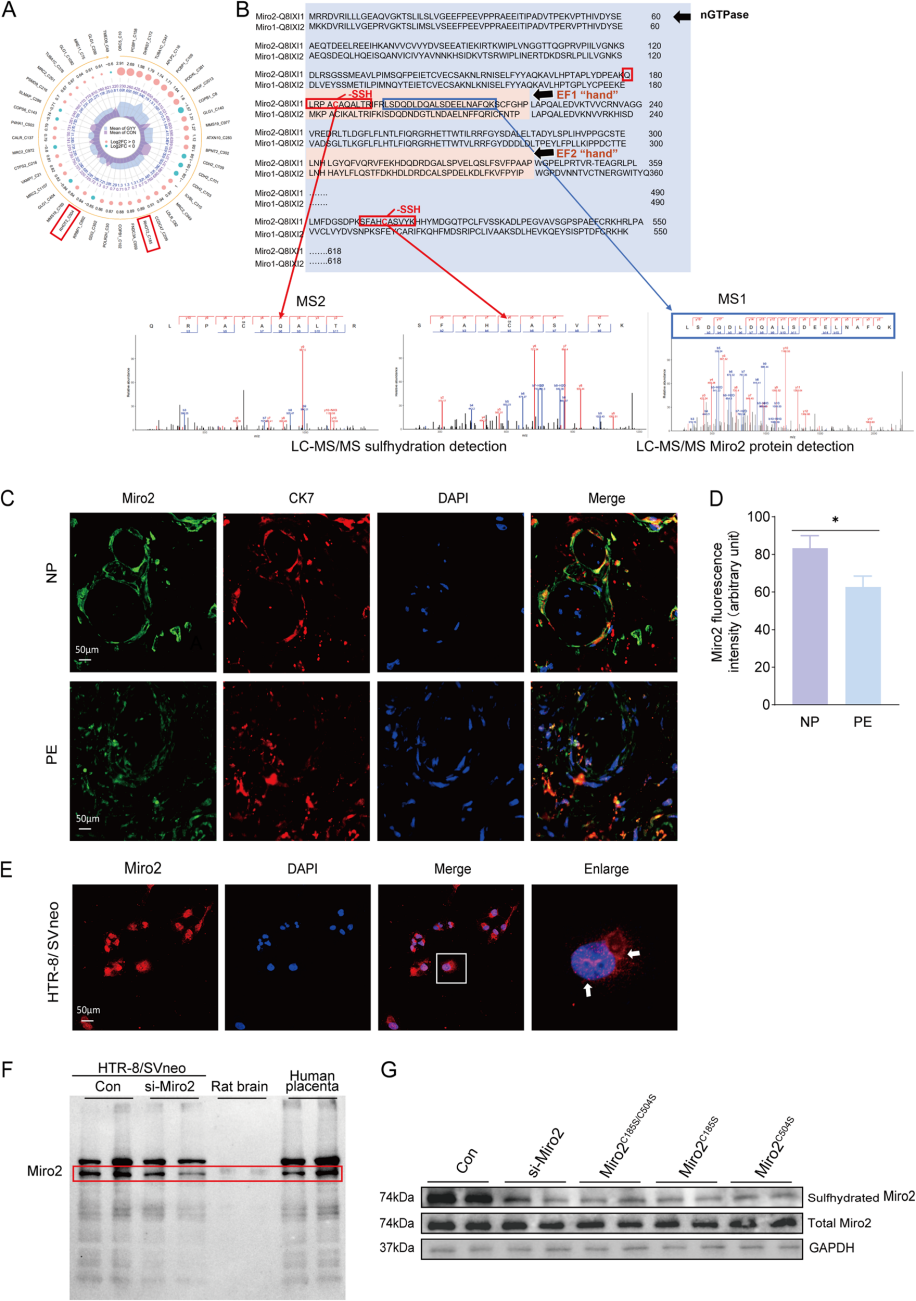
Miro2 expression on human cytotrophoblast and HTR-8/SVneo cell line. **A** The top 45 differentially sulfhydrated proteins between Control and GYY4137 groups in HTR-8/SVneo. RHOT2, also named Miro2, showed C504 and C185 were highly sulfhydrated (red rectangle). **B** The Miro1 and 2 protein sequence blasted on Uniprot. The mass spectrometry (MS) showed the detected sites (MS2, -SSH detection) and sequence (MS1, peptide detection,) for Miro2. **C**, **D** Immunofluorescent staining with anti-Miro2 and anti-CK7 antibodies. CK7 positively stained cells were partially Miro2 positive in human placenta slices (white arrow). Bar graph analyzed the fluorescent intensity of Miro2 in placenta from PE and NP. *n* = 6 each group. **E** Fluorescent staining of Miro2 in HTR-8/SVneo cells. **F** Western blot detected Miro2 protein in HTR-8/SVneo and rat brain extract. Brain Miro2 was taken as the positive control, and a similar protein band with anti-Miro2 antibody was detected in HTR-8/SVneo (red rectangle). A designed si-RNA targeting Miro2 was applied to test the band specificity of Miro2. **G** Western blot detection of sulfhydrated Miro2 in control, si-Miro2 and mutant Miro2 groups in HTR-8/SVneo. *P < 0.05. PE preeclampsia, NP normal pregnancy.

Since the CBS/H_2_S signal on mitochondria improved mitochondria dynamics, the underlying mechanism is still obscure. Physiologically, H_2_S exerts its bioactivity partly via protein sulfhydration which is not well studied in pregnancy and pregnancy related diseases. Recent research reported a pregnancy-dependent sulfhydration that informed novel roles of protein sulfhydration [15] implying the physiological significance of protein sulfhydration in pregnancy physiology. Via protein mass spectrum (MS) and protein post-translational modification, 828 sulfhydrated proteins with differential significance in the GYY4137 treated group versus the control group were enriched subcellularly (Fig. S3O, Table S2). These sulfhydrated proteins were mainly enriched in cytoplasm (35.38%) and nucleus (28.4%) (Fig. S3O). There are 90 differentially sulfhydrated proteins involved in GYY4137 treated cell with 80 proteins upregulated and 12 downregulated enriched in mitochondria (Fig. S3N). The top 45 sulfhydrated proteins (Table S3) were shown as the radar graph in Fig. 6A in which only protein RHOT2 (also called Miro2) was sulfhydrated at two cysteine sites, C185 and C504 with 1.85- and 1.79-fold increase respectively (Fig. 6B). Interestingly, Miro2 is responsible for mitochondria transporting in the neuron axon. We then tested if Miro2 contributed to mitochondria distribution and functions in the cytotrophoblast which is the first report so far.

**Fig. 7:**
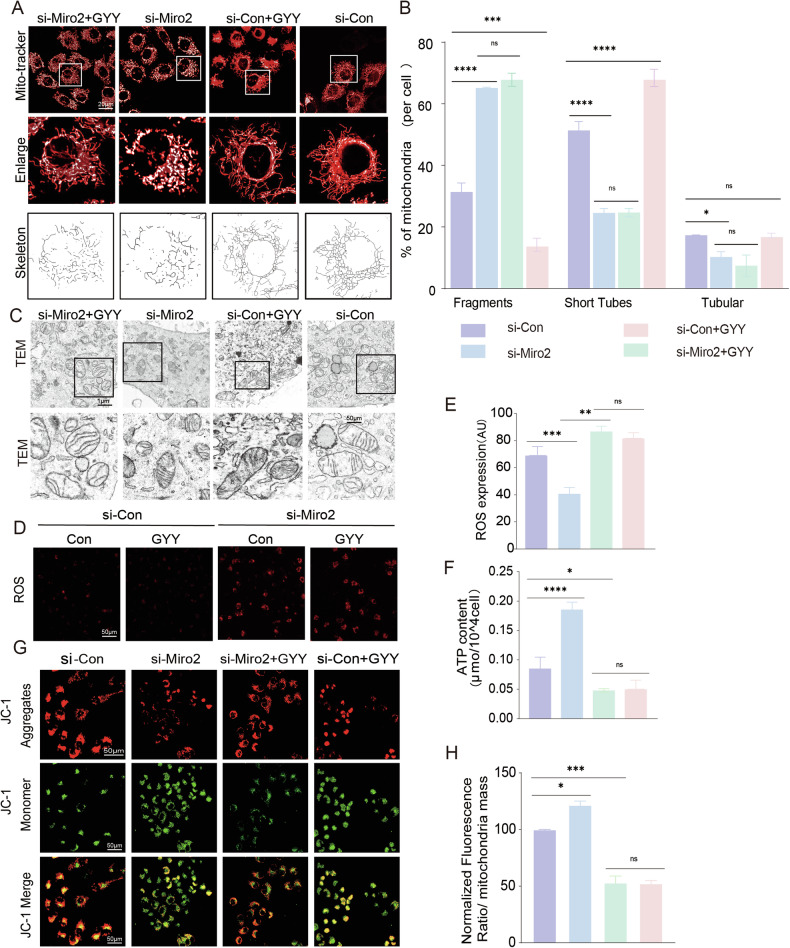
Miro2 played a role in mitochondria functions. **A**, **B** Live-cell mitochondria tracking in si-Control, si-Control+GYY4137, si-Miro2 and si-Miro2 + GYY4137 groups. Mitochondria were shorter in si-Miro2 and si-Miro2 + GYY4137 versus si-Control separately. Statistical bar graph analyzed the mitochondria fragments, short tubes and tubular in si-Control, si-Miro2 and si-Miro2 + GYY4137 groups. *n* = 12 each group. **C** TEM images showed mitochondria size, shapes and cristae in si-Control, si-Control+GYY4137, si-Miro2 and si-Miro2 + GYY4137 groups. Mitochondria size and shapes were relatively even and regular in the three groups. Cristae was less clear in si-Miro2 and si-Miro2 + GYY4137 groups. ‘M’: mitochondria. **D**, **E** ROS detection with fluorescent detector (red) in si-Control, si-Control+GYY4137, si-Miro2, and si-Miro2 + GYY4137 groups. Bar graph showed the statistical difference between groups. *n* = 7 each group. **F** ATP content tested in si-Control, si-Control+GYY4137, si-Miro2, and si-Miro2 + GYY4137 groups. **G**, **H** Δψm was assessed using JC-1 fluorescent detector (red in aggregates, green in monomer). The bar graph showed normalized fluorescent ratio of red/green to indicated Δψm variance in si-Control, si-Miro2 and si-Miro2 + GYY4137 groups. *n* = 7 each group. ‘ns’: no significance; *P < 0.05; **P < 0.01; ***P < 0.001; ****P < 0.0001.

The immunofluorescent data showed Miro2 was positively stained in both invasive trophoblast of human placenta (Fig. 6C, D) and HTR-8/SVneo cells (Fig. 6E). Western blot detected Miro2 protein in HTR-8/SVneo extract with brain lysate as positive control (Fig. 6F). The fluorescence intensity was decreased in PE placenta (Fig. 6D). To evaluate if Miro2 and the sulfhydration sites were involved in mitochondria dynamic and functional changes, we designed Miro2 siRNA (si-Miro2) and Miro2^C185/C504^ mutations, and transfected si-Miro2/mutations into HTR-8/SVneo cells. The Miro2 sulfhydration was detected using Dimedone switch method (Fig. 6G). Point mutation of C185 and/or C504 to serine (C185S, C504S) (Table S4) decreased Miro2 sulfhydration profoundly (Fig. 6G), suggesting C185 and C504 were the key sulfhydration sites of Miro2. The knockdown efficiency of Miro2 was shown in Fig. S4E and the rescue experiments were performed to check the si-Miro2 knockdown efficiency (Fig. S6). Morphologically, mitochondria short tubes increased and tubular length decreased significantly following si-Miro2 transfection (Fig. 7A, B). However, GYY4137 didn’t recover si-Miro2 induced morphological changes (Fig. 7A, B). TEM study showed no significant variation of mitochondria numbers and length/width ratio in si-Control, si-Miro2 and si-Miro2 + GYY4137 groups (Fig. 7C, Fig. S4A, B). Mitochondria cristae was not as much as si-Control group (Figs. 7C and S6C). These data indicated Miro2 didn’t alter the basal mitochondria structure but Miro2 knockdown enhanced mitochondria fragmentation and prevented GYY4137-induced tubular elongation and short tubes formation. Meanwhile, ROS production increased and ATP content decreased in the si-Miro2 group (Fig. 7D–F). Δψm were reduced following Miro2 knockdown in si-Miro2 and si-Miro2 + GYY4137 groups (Fig. 7G, H).

**Fig. 8:**
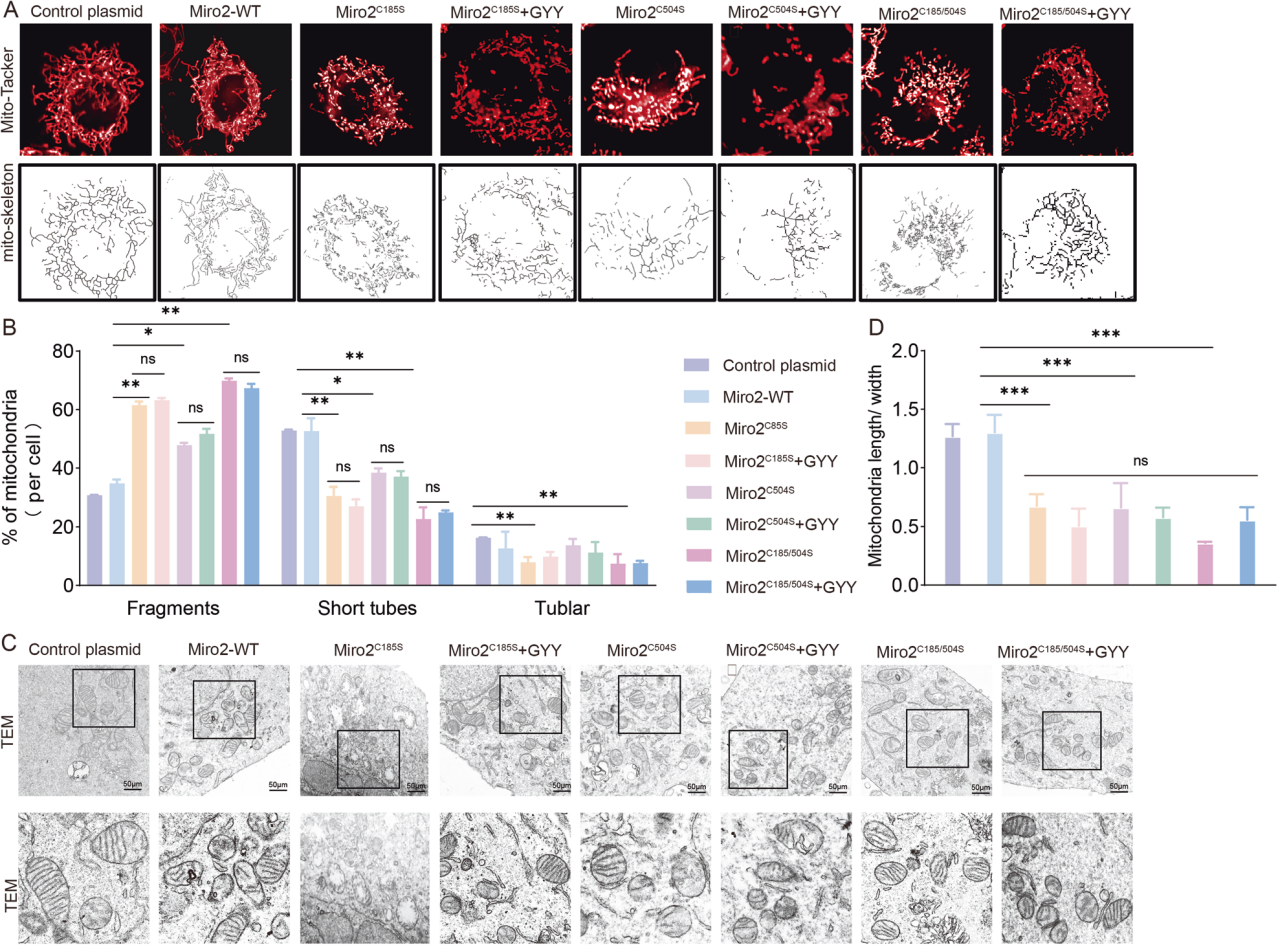
Miro2^C185/C504^ are the regulatory sites in modulating mitochondria morphology. **A**, **B** Live-cell mitochondria tracking in Control plasmid, Miro2-WT plasmid, Miro2^C185S^, Miro2^C504S^, Miro2^C185S/C504S^ with or without GYY4137 groups. Mitochondria tubular was shorter in Miro2^C185S^ and Miro2^C185S/C504S^ with and without GYY4137 groups versus Control plasmid separately. Statistical bar graph analyzed the mitochondria fragments, short tubes and tubular in the 8 groups. *n* = 12 each group. **C**, **D** TEM images showed mitochondria size, shapes and cristae in Control plasmid, Miro2-WT plasmid, Miro2^C185S^, Miro2^C504S^, Miro2^C185S/C504S^ with or without GYY4137 groups. Mitochondria size and shapes were relatively even and regular in Control and Miro2 plasmid groups. Mitochondria size was uneven in Miro2^C185S^, Miro2^C504S^, Miro2^C185S/C504S^ with and without GYY4137 groups. *n* = 9 each group. ‘ns’: no significance; *P < 0.05; **P < 0.01; ***P < 0.001; ****P < 0.0001.

**Fig. 9:**
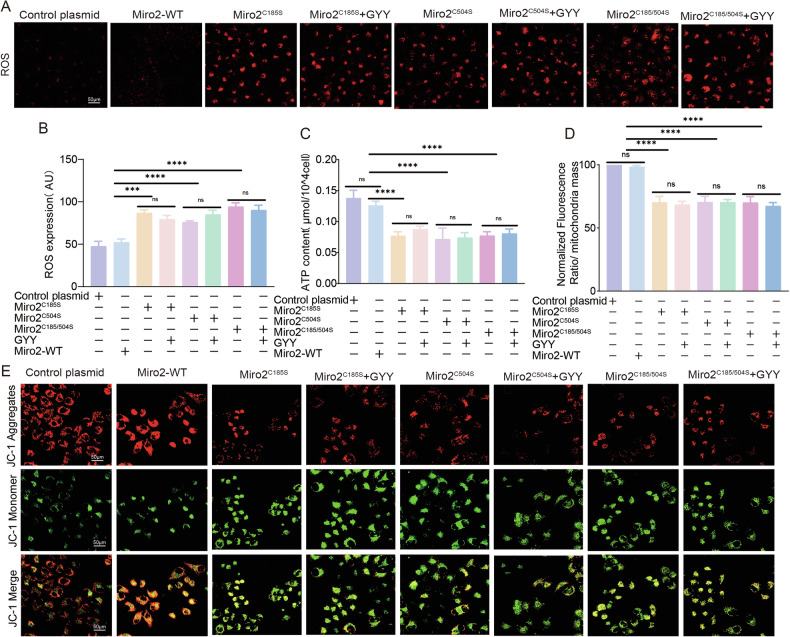
Miro2^C185/C504^ regulates mitochondria functions. **A**, **B** ROS detection with fluorescent detector (red) in Control plasmid, Miro2-WT plasmid, Miro2^C185S^, Miro2^C504S^, Miro2^C185S/C504S^ with or without GYY4137 groups. Bar graph showed the statistical difference between groups. *n* = 7 each group. **C** ATP content tested in Control plasmid, Miro2-WT plasmid, Miro2^C185S^, Miro2^C504S^, Miro2^C185S/C504S^ with or without GYY4137 groups. **D**, **E** Δψm was assessed using JC-1 fluorescent detector (red in aggregates, green in monomer). The bar graph showed normalized fluorescent ratio of red/green to indicated Δψm variance in Control plasmid, Miro2-WT plasmid, Miro2^C185S^, Miro2^C504S^, Miro2^C185S/C504S^ with or without GYY4137 groups. *n* = 7 each group. ‘ns’: no significance; *P < 0.05; **P < 0.01; ***P < 0.001; ****P < 0.0001.

To test if sulfhydration plays a role in regulating mitochondria, we mutated Miro2-C185 or C504 to serine (Miro2^C185S^, Miro2^C504S^), or double mutation of C185 and C504 to serine (Miro2^C185S/C504S^) simultaneously. Transfecting HTR-8/SVneo with Miro2^C185S^, Miro2^C504S^, and Miro2^C185S/C504S^ plasmid separately increased fragmentation and decreased short tubes significantly (Fig. 8A, B). GYY4137 couldn’t rescue Miro2 mutation/s induced mitochondria fragmentation and short tubes (Fig. 8A, B) suggesting Miro2^C185^ and Miro2^C504S^ were mostly involved in the fragmentation and short tubes formation. The TEM study showed mitochondria numbers and length/width ratio were similar in Control plasmid, Miro2^C185S^, Miro2^C504S^, and Miro2^C185S/C504S^ with or without GYY4137 transfection groups (Figs. 8C, D, and S4C, D). The mitochondria shapes in Miro2^C185S^, Miro2^C504S^, and Miro2^C185S/C504S^ with or without GYY4137 groups were not as uniform as those in Control plasmid treated group, and mitochondria were large in size with less cristae (Fig. 8C). Increased mitochondria ROS level were observed in Miro2^C185S^, Miro2^C504S^ and Miro2^C185S/C504S^ plasmid treated group comparing with Control plasmid group, while GYY4137 had no effect on Miro2^C185S^, Miro2^C504S^ and Miro2^C185S/C504S^ double mutant plasmid treated cells comparing with no GYY4137 treated ones (Fig. 9A, B).

Decreased ATP production and ATP linked production were observed in si-Miro2, Miro2^C185S^, Miro2^C504S^, and Miro2^C185S/C504S^, while GYY4137 didn’t alter si-Miro2, Miro2^C185S^, Miro2^C504S^, and Miro2^C185S/C504S^ induced ATP content reduction (Fig. 9C, Fig. S5A–C). Δψm were decreased in both Miro2^C185S^ and Miro2^C185S^ + GYY4137 groups (Fig. 9D, E) indicating GYY4137 couldn’t protect mitochondria in Miro2 mutants’ cells.

To further confirm the cellular locations of Miro2, CBS and mitochondria, immunofluorescent staining targeting Miro2, CBS and Tom-20 (mitochondria marker) was processed and the data showed Miro2, CBS and Tom-20 colocalized partially in HTR-8/SVneo although not all (Fig. S4F).

### Miro2^C185/C504^ on cytotrophoblast invasion and migration

**Fig. 10:**
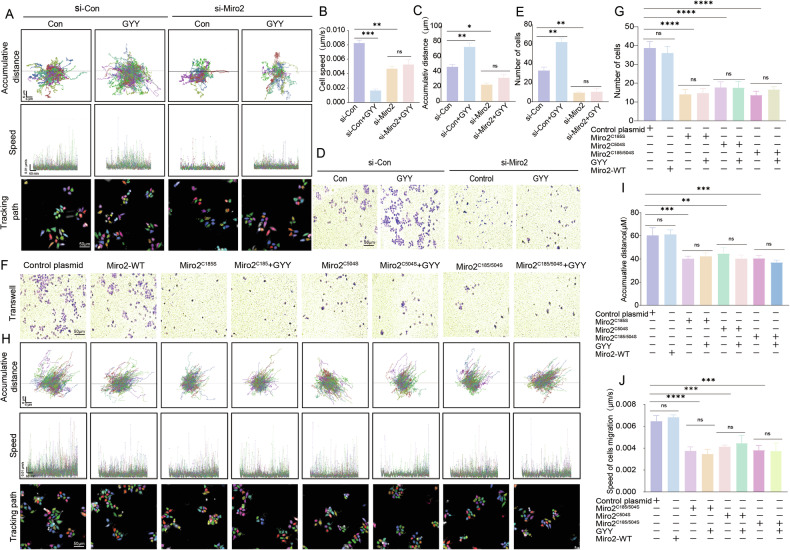
Miro2^C185/C504^ in HTR-8/SVneo migration and invasion. **A**–**C** Live-cell tracking distance and speed were recorded and analyzed with confocal microscope in si-Control, si-Control+GYY4137, si-Miro2, and si-Miro2 + GYY4137 groups. The accumulative distance and moving speed were analyzed between groups. *n* = 8 each group. **D**, **E** Transwell study to analyze the penetrated HTR-8/SVneo trophoblast numbers staining with crystal violet in si-Control, si-Control+GYY4137, si-Miro2, and si-Miro2 + GYY4137 groups. Bar graph showed the statistical differences between groups. *n* = 7 each group. **F**, **G** Transwell study to analyze the penetrated HTR-8/SVneo trophoblasts in Control plasmid, Miro2-WT plasmid, Miro2^C185S^, Miro2^C504S^, Miro2^C185S/C504S^ with or without GYY4137 groups. Bar graph showed the statistical differences between groups. *n* = 7 each group. **H**–**J** Live-cell tracking distance and speed were recorded and analyzed with confocal microscope. The accumulative distance and moving speed were analyzed between groups. *n* = 7 each group. ‘ns’: no significance; *P < 0.05; **P < 0.01; ***P < 0.001; ****P < 0.0001.

To analyze the functional significance of Miro2 on cytotrophoblast migration and invasion, we performed live-cell tracking study and Transwell-test using the HTR-8/SVneo cell line. live-cell tracking data showed that the accumulative distance and speed were increased in GYY4137 treated si-Control and Control plasmid groups compared with Control groups without GYY4137 (Fig. 10A–C). si-Miro2 decreased the accumulative distance and speed compared with si-Control group (Fig. 10A–C). GYY4137 treatment didn’t alter si-Miro2 induced down regulation of accumulative distance and speed (Fig. 10A–C). Miro2^C185S^, Miro2^C504S^, and Miro2^C185S/C504S^ groups showed decreased accumulative distance and speed, while GYY4137 couldn’t rescue the reduced accumulative distance and speed (Fig. 10F lower 3 panel, H and I). Meanwhile, the transwell data showed that si-Miro2 decreased penetrated cell numbers in si-Miro2, Miro2^C185S^, Miro2^C504S^, and Miro2^C185S/C504S^ groups while GYY4137 enhanced cell penetrating through the transwell in si-Control and Control plasmid groups but had no effect on si-Miro2, Miro2^C185S^, Miro2^C504S^, and Miro2^C185S/C504S^ double mutant groups (Fig. 10D, E, F upper panel, and G). These data indicated Miro2^C185^/^C504^ were the key regulatory sites for HTR-8/SVneo cell invasion and migration.

## Discussion

Multiple bio-factors regulate cytotrophoblast invasion and migration [16–18] which are vital processes in spiral artery remodeling [18], and the successful remodeling of spiral artery incorporated with the invaded cytotrophoblast is the last, crucial step in maintaining healthy pregnancy [18, 19]. In this study, we described the new intrinsic pathway regulating mitochondria and thus promoting cytotrophoblast invasion and migration: 1) CBS/H_2_S translocation onto mitochondria physiologically; 2) CBS derived H_2_S regulated mitochondria homeostasis via Miro2^C185/C504^ sulfhydration; 3) mitochondria homeostasis determines cytotrophoblast invasion and migration in vitro.

The bioactivity of H_2_S is diverse, and its physiological significance is far from been discovered in healthy people or diseases in human. Studies from clinical samples show that H_2_S reduction is observed in preeclampsia (PE) [20] and intra-uterus growth restriction (IUGR) [21]. Reducing systemic H_2_S production confirmed the unfavorable impact on animal pregnancy [22] indicating the physiological significance of H_2_S in pregnancy and pregnancy related diseases. However, the previous studies on H_2_S focused on the vascular compartment, less is known about whether the fetus compartment receives H_2_S regulation and the physiological significance needs to be discovered.

In 2020, our team reported that trophoblast H_2_S play vital roles in maintaining early pregnancy [5], while leaving the significance and mechanisms of H_2_S on trophoblast invasion unanswered, although we have observed H_2_S enhanced trophoblast invasion since then. A recent report also confirmed the phenomenon that exogenous H_2_S promoted cytotrophoblast migration and invasion [23] without knowing the underlying mechanisms. Meanwhile, exogenous H_2_S was reported to be protecting cytotrophoblast (HTR-8/SVneo) apoptosis via anti-oxidative mitochondria damage recently [24]. All the above evidence suggests exogenous H_2_S is an important regulator of trophoblast invasion and survival. We reported the same observation in this study. Exogenous H_2_S indeed promoted trophoblast invasion and migration. But, human cytotrophoblast themselves also synthesize and produce CBS and H_2_S as shown in Fig. S1G. Knocking down CBS by siRNA decreased H_2_S content in HTR-8/SVneo (Fig. S1F) indicating trophoblast produced H_2_S at least partially via CBS. CBS produced H_2_S contributed largely to the HTR-8/SVneo invasion and migration. Interestingly, this endogenous CBS/H_2_S mediated cell invasion and migration is mitochondria dependent. While, exogenous H_2_S facilitated CBS/H_2_S translocation onto mitochondria which was on the upstream side of the CBS/H_2_S signal pathway. This finding has only been reported in cytotrophoblast as far.

Another finding is that, the H_2_S donors (GYY4137 and Na_2_S) used in this study are within 5 µM in concentration which was largely lower than that in other studies [23, 25] (mostly higher than 100 µM). Although, the exact tissue concentration of H_2_S has been in debate for a long time due to the lack of precise H_2_S detectors or methods. The modified monobromobimane (MMBB) method has reported 0.7-3 µM sulfide in mammalian plasma [26, 27] and 15 nM in mouse plasma [28]. In this case, the H_2_S concentration we used in this study is close to the physiological H_2_S levels which have rarely been reported before. Thus, the enhanced invasion and migration of cytotrophoblast in response to low dose H_2_S is a significant and new finding that brings new insights in understanding the regulatory profiles of cytotrophoblast. Since the cytotrophoblast synthesize and produce H_2_S by themselves, physiologically, the cytotrophoblast may receive regulation by H_2_S in a para or autocrine manner.

Mitochondria, the vital organelle to full fill energy demand and cell signaling in maintaining cell survival and functions, is known to be essential in the extravillious cytotrophoblasts invasion [29, 30]. Dysregulation of mitochondria contents, structure and function was identified as significant contributor to the pathophysiology of preeclampsia (PE) [30]. Improving mitochondrial functions has been given full consideration for the therapeutic strategies of PE [31, 32]. In consistence with the previous report [33], we observed the protective effect of low-dose H_2_S on mitochondria functions. Different from the previous report that H_2_S donate electron during mitochondria respiration, we found that the low-dose H_2_S preserved mitochondria functions via promoting CBS translocation onto mitochondria which is new to our knowledge. We think the two mechanisms are hybrid in cells, or if not, are due to the locations of H_2_S in subcellular organelles. After CBS translocation, the local synthesized H_2_S aggregated around mitochondria and sulfydrated cysteine residues of proteins in HTR-8/SVneo, in which mitochondrial Rho GTPase 2 (Miro2) was included. Miro 1/2 are the outer mitochondrial membrane proteins which usually mediates mitochondria transport and inter-mitochondria communication within cells [34–36]. Miro 1 plays vital roles in neuronal diseases [34] while Miro2 is a key regulator of inter-mitochondria communication outside the nervous system [36]. Both Miro1 and 2 proteins were detected in HTR-8/SVneo extracts, but only Miro2^C185/C504^ were highly sulfhydrated and were involved in mitochondria mediated cell invasion and migration. This finding indicates that Miro2 is the new regulatory protein on mitochondria functions. However, the functional ratio of Miro2 on mitochondria functions and cell invasion versus other compartments needs further evaluations in the future. Altogether, despite the different cellular mechanisms mediated by H_2_S, we believe that low dose H_2_S enhances mitochondria functions and finally promotes cell bioactivity. Meanwhile, 200 µM NaHS exposure is cell toxic in mouse heart mitochondria and a sublethal dose of H_2_S gas induced substantial oxidative stress in mice [37] indicating the cell toxicity induced by high dose H_2_S both in vitro and in vivo. Our unpublished data also observed the toxicity of a 100 µM H_2_S donor on HTR-8/SVneo. Thus, these data raise the feasibility of H_2_S and H_2_S donors as candidate therapeutics for PE and mitochondria related diseases in pregnancy.

H_2_S synthetic enzyme translocation onto mitochondria to sustain mitochondria functions is not solely the focus of this study. CSE is translocated onto mitochondria in smooth muscle cells to preserve ATP production under hypoxia [11]. In this study, CBS/H_2_S translocation onto mitochondria happens in unstressed HTR-8/SVneo trophoblasts in response to low dose H_2_S donor. Similar to the previous report [13], we found CBS/H_2_S translocation onto mitochondria protected mitochondria functions via enhancing ATP production, decreasing ROS release and maintaining mitochondria membrane potential simultaneously. However, we didn’t observe the mitochondrial destruction of trophoblast in the long-term exposure to H_2_S (48 hours in this study) as reported [13]. This might be due to H_2_S donor concentrations and different cell lines we used. NaHS at 100 µM was used in other studies while GYY4137/Na_2_S at less than 5 µM was used in our study. In our unpublished data, we found GYY4137/Na_2_S/NaHS led to cell death at concentrations of more than 50 µM indicating trophoblast might be more sensitive to H_2_S stimuli. And, the other trophoblast cell line BeWo cells, which is of carcinoma origin, showed no response to low dose GYY4137 (data not shown here). We confirmed that HTR-8/SVneo trophoblasts act differently from the other cell lines especially the cancer cell lines.

In addition, data from this study showed that CSE and MST3 were also detectable in human placenta and CTB cell line HTR-8/SVneo cells, while only CBS was expressed the most among the three enzymes, and CBS was decreased significantly in placenta tissues from PE patients compared with the normal pregnancy group which is consistent with previous report [10, 38].

**Fig. 11:**
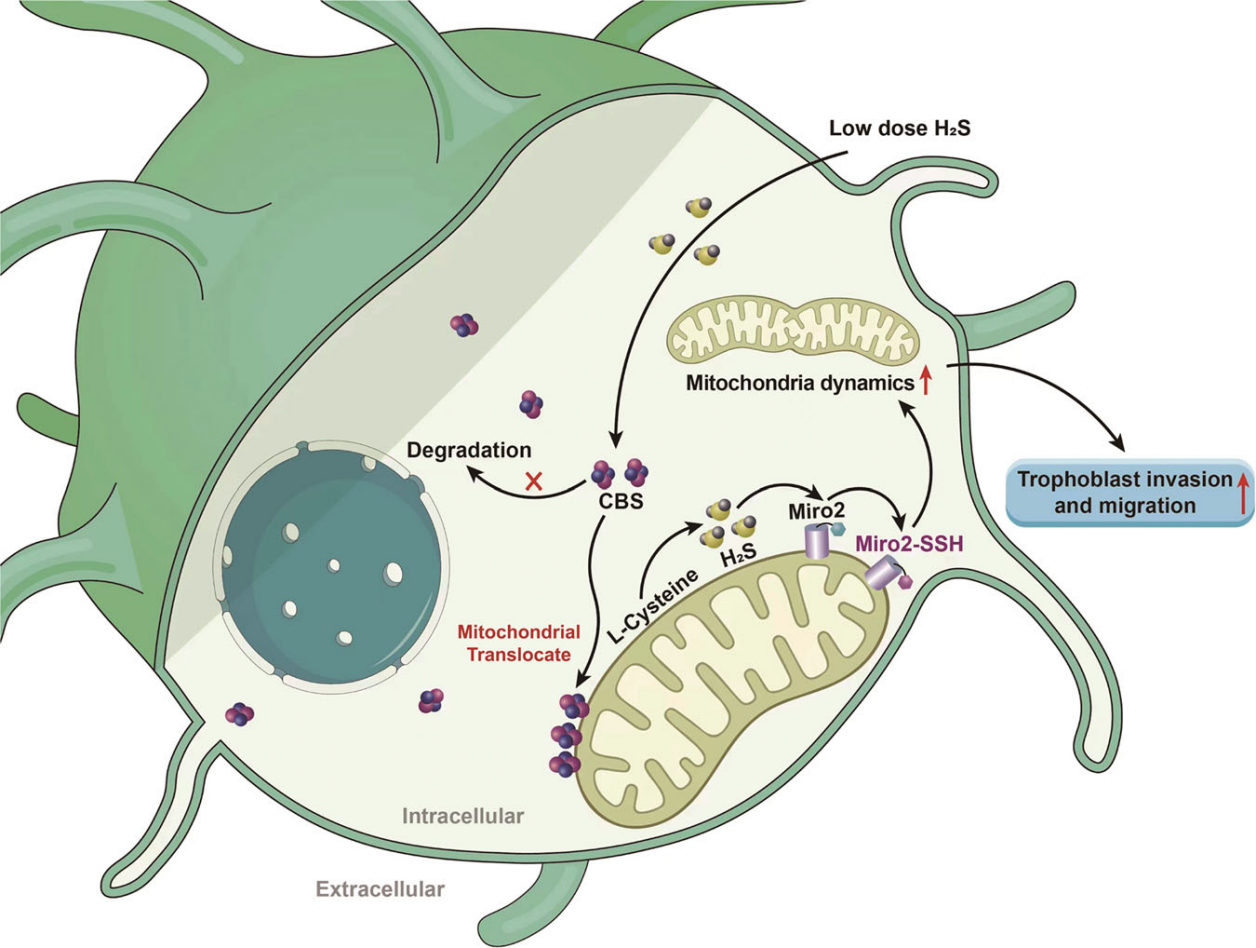
Schematic diagram of low dose H_2_S on human trophoblast invasion and migration. Low dose H_2_S stimulates CBS translocation onto mitochondria and inhibited CBS degradation simultaneously. The CBS aggregation on mitochondria catalyze L-cysteine to synthesize H_2_S in situ. Mitochondria CBS/H_2_S sulfhydrated Miro2-C185 which facilitated mitochondria dynamics, and the latter enhanced trophoblast invasion and migration consequently.

In conclusion (Fig. 11), human cytotrophoblast receives low dose H_2_S regulation with enhanced invasion and migration as consequences; CBS/H_2_S sustained mitochondria functions via Miro2^C185/C504^ sulfhydration. These findings established a new regulatory pathway for cytotrophoblast invasion and migration. The beneficial effect by low dose H_2_S application to cytotrophoblast implies H_2_S is a plausible candidate to rescue the reduced CBS expression in PE patients which is of translational significance.

## Materials and methods

### Human placenta tissues

The maternal-fetus interface placenta tissues were obtained from 10 normal pregnancies and 10 severe pre-eclampsia patients who received cesarean section in the First Affiliated Hospital of Shandong First Medical University. Severe pre-eclampsia was diagnosed clinically following the ACOG guideline (2020). The maternal-fetus interface parts were collected for western blot and immunofluorescence study. The sample size was determined by power analysis based on pilot experiments (significance level 0.05, power 80%). The human sample study was approved by the Human Research Ethics Board of the First Hospital affiliated to Shandong First Medical University, and all participants were given informed consent before being enrolled in the study. The ethical No is KYLLTZH-2023-011.

### H_2_S detection

The human plasma was collected via centrifuging the peripheral circulating blood at 3000 rpm for 15 minutes. H_2_S content was detected using two methods: the modified Methylene blue method [26], and the commercially available H_2_S detecting kit (A146-1-1, Nanjing Jiancheng Bioengineering Institute) [39–42].

The modified Methylene blue method: In brief, Zn^2+^ was added to plasma samples to deposit H_2_S, HS^−^ and S^2−^, as well as plasma protein, and then NaOH was used to re-dissolve plasma protein. ZnS deposition was re-dissolved by the addition of N, N-dimethyl -p-phenylenediamine, and the remnant protein was deposited by trichloroacetic acid. After centrifugation, ferriammonium sulfate was added to the supernatant fluid to generate methylene blue, which was analyzed by a spectrophotometer at 665 nm.

H_2_S detecting kit: blood samples were prepared and tested following the instructions provided by the company. At least 3 technical replications were tested for each sample.

### Western Blot

Tissue protein preparation: 100 mg of maternal-fetus placental tissue were collected and extracted with 1 ml of lysate with PMSF at 4 °C. After being homogenized with a homogenizer (KZ-5F-3D, Servicebio), samples were centrifuged at 12000 g for 20 minutes, 4 °C. The supernatants were collected for protein detection.

Whole cell protein preparation: HTR-8/SVneo cells of 90% full were collected for protein extractions. Wash cells with cold PBS for 3 times and add RIPA buffer (P 0013, Vazyme) to break cells on ice for 30 minutes. Collect the cells and centrifuge at 12000 g for 20 minutes, 4 °C. The supernatants were collected for protein detection. The total protein concentrations of all groups were determined using a BCA protein assay kit (E112-02, Vazyme).

Mitochondrial protein preparation: The Mitochondria Isolation Kit (C3601, Beyotime) was used to extract mitochondria from HTR-8/SVneo cells following the manufacturer’s instructions. In brief, collect cells after digestion with trypsin, and centrifuge at 200 g at room temperature. Suspend cells with cold PBS and centrifuge at 600 g for 5 minutes at 4 °C. Add the isolation reagent with PMSF from the kit to suspend cells and culture for 15 minutes on ice. Homogenize for 10-30 times using a homogenizer and centrifuge at 1000 g for 10 minutes at 4 °C. Keep the supernatant to centrifuge again at 11000 g for 10 minutes at 4 °C. The precipitation contained mitochondria and supernatant contained cytosol proteins. Mitochondria lysis buffer and RIPA buffer with PMSF were added to the precipitation and supernatant separately for mitochondria and cytosol protein collection.

The total proteins (20-30 µg) were loaded on 10% polyacrylamide SDS gels to separate target proteins. Then transfer proteins from the SDS gel onto PVDF membranes. Then block the PVDF membrane in a 5% skimmed milk solution for 1 h. Incubate the membranes with primary antibodies anti-CBS (AB-226194, Abcam), anti-CSE (AB-136604, Abcam) anti-MST3 (PA5-51548, Thermo), anti-TOM20 (YT4696, Immunoway) and anti-GAPDH (AB-646 13063, Abcam) at 4 °C overnight. The second day, incubate membranes with the secondary HRP-conjugated goat anti-rabbit/ mouse IgG (AB181662, Abcam) for 1 h at room temperature. The enhanced chemiluminescence (ECL) was used to detect protein bands and Image-J software was used to analyze the bands density.

All the original western blots were included in Supplemental Material- Western Blots.

### Sulfhydration protein detection [43, 44]

HTR-8/SVneo cells were seeded in 10 cm culture dishes and treated with vehicle solution or GYY4137 for 48 hours. On the day of harvesting, cells were washed three times with cold PBS. Then diluted NBD-Cl (163260, Sigma-Aldrich) with 150 µl of RIPA and PMSF (PMSF: RIPA = 1:10) into working concentration of 5 mM, add the mix to plates and scrub the cells. Incubated cells at 4 °C for 30 minutes. Then add NBD-Cl to a final concentration of 15 mM, and incubate for another 30 minutes. Collect proteins using a OrgoSol-PROTEIN-Concentrate (TM) kit (C006125, Sangon Biotech), vacuum drying the proteins for 10-15 minutes and resolve proteins with 200 µl of PBS (50 mM, Ph=7.4) with 2% SDS. A volume of 20 µl was used for protein concentration detection and input loading. Then add DCP-Bio1 (NS1226, Sigma-Aldrich) to the rest of the proteins to a final concentration of 50 µM. Incubate the proteins for 1.5 hours at 37 °C. Precipitate proteins again with the OrgoSol-PROTEIN-Concentrate (TM) kit, vacuum drying the proteins for 10-15 minutes and resolve proteins with 50 mM PBS with 0.1% SDS. Incubate protein solutions with Pierce™ Streptavidin Magnetic Beads (88816, ThermoScientific) at 4 °C overnight with rotation. The next day, wash the beads with PBS for three times, and boil the proteins in loading buffer at 95 °C for 10 minutes. Samples were ready for Western blot analysis. Sulfhydrated proteins were detected with an anti-Miro2 antibody and the total Miro2 proteins as the loading control.

### Immunofluorescence

HTR-8/SVneo cells were seeded on coverslips and fixed with 4% paraformaldehyde fix solution (30525-89-4, Macklin) for 30 minutes. Staining of CBS/CSE/MST3 in HTR-8/SVneo were processed after labeling cells with Mito-Tracker (C10498, Beyotime) in 96-well microplate before fix. Penetrate the cell membrane with 0.1% triton X-100 for 10 minutes. Block the coverslips or microplate with 1% BSA at room temperature for 45 min and incubate the cells with primary antibodies at 4 °C overnight. After washing, the cells three times with PBS, the cells were incubated with a secondary Alexa 488-conjugated goat anti-rabbit IgG (H + L) antibody (AB 150077, Abcam) for 1 h at room temperature. Fluoroshield Mounting Medium with DAPI (AB 104139, Abcam) was used to mark nuclei after 3 times washing with PBS. Immunofluorescence signals were visualized by confocal microscopes (Ti2-S-HU, Nikon; Operetta CLS, PerkinElmer).

Human placenta tissues were collected and fixed with 4% paraformaldehyde. After embedding tissues in paraffin, the samples were sectioned into 2-5 µm slices. After dewaxing and hydration, the slices were heated in 0.01 M citric acid buffer (005000, Thermo Fisher Scientific) for antigen retrieval. Block the slices with 5% BSA at room temperature for 1 h, and then, incubate the slices with primary antibodies against Cytokeratin 7 (CK-7) (17513-1-AP, Proteintech) at 4 °C overnight. After washing with PBS, the slices were incubated with Alexa 640-conjugated goat anti-rabbit IgG (H + L) antibody (AB 150079, Abcam) for 1 h at room temperature. Wash the slices with PBS for 3 times, block the slices with 5% BSA at room temperature for 1 h again. Repeat the primary and secondary antibody incubations again and mount the slices with DAPI (AB 104139, Abcam) for microscopy imaging (Ti2-S-HU, Nikon). The primary antibodies: anti-CBS、anti-CSE、anti-MST3；anti-GPER (AB 260033, Abcam) anti-Miro-2 (AB 224089, Abcam) were used. Alexa 647-conjugated goat anti-rabit IgG (H + L) antibody (AB150083, Abcam), and Alexa 488-conjugated goat anti-rabbit IgG (H + L) antibody (AB150077, Abcam) were used.

### q-PCR technique

Total cell RNA was extracted using the RNA-easy™ Isolation Reagent (Q712-02, Vazyme), and reverse transcription was performed using the PrimeScript ™ RT kit (P 611-01, Vazyme). The relative mRNA levels were measured using a Bio-Rad iQ 5 Multicolor Real-Time polymerase chain reaction (RT-PCR) system (1725122, Bio-Rad Laboratories,). Relative CBS gene expression was measured using the 2 − ΔΔCt method and normalized to the level of L19. Primer sequences were in Table S1.

### Cell culture

HTR-8/SVneo cell lines (HTR-8/SVneo) were purchased from ATCC. The STR analysis and mycoplasma contamination test were done at the end of the experiments. Cells were cultured with DMEM (12800017, Thermo Fisher Scientific) supplemented with 10% fetal bovine serum (BS-1105, OPCEL, Inner Mongolia Opcel Biotechnology Co.,Ltd.), 100 U/mL penicillin and 100 g/mL streptomycin (15070063, Thermo Fisher Scientific) at 37 °C, 5% CO_2_, and saturated humidity. A 0.25% concentration of trypsin (25200072, Thermo Fisher Scientific) was used to digest logarithmic phase cells for the experiment. A suspension of individual cells was prepared and seeded into an appropriate plate. All the cells were treated with GYY4137 (1 µM) for 24 hours in each experiment unless specified otherwise.

Cycloheximide (CHX, 200 µg/ml) was added into the cells to inhibit protein synthesis. And the protein levels were detected accordingly at 0, 4, 8, 12, and 24 hours in this study to assess the protein stability.

### siRNA transfection

Prepare cells in 6-well plates, and transfect cells with the corresponding siRNAs using Lipo 8000 reagent (C0533, Beyotime). After 48 hours of transfection, collect the cells for the transfection efficiency test using western blot. A 50% reduction of protein was considered to be knocked down efficiently. si-RNA sequences were in Table S1.

### Cell invasion and migration study

Transwell study was used to assess cell invasion. The transwell for invasion is a commercially available Biocoat Matrigel Invasion Chamber kit (354480, Corning) with 8.0 µm PET membrane in 24-well plates. A concentration of 3 × 10^3^cells/well was seeded into the upper transwell filled with 200 µL FBS-free DMEM. The lower chambers were filled with 800 µL DMEM containing 10% FBS. After 48 h incubation, a tipped swab was used to remove inner side cells leaving the outside cells in situ for staining. Crystal violet (C0121, Beyotime) was used to stain the cells. For each group, at least three independent wells were included, and five to six photographs were taken for each group, and the results were analyzed using Image J.

Cell migration was evaluated by analyzing live cell tracking properties. Cells were seeded at a concentration of 2500 cells per well in a 96-well CellCarrier Ultra plate (6055308, PerkinElmer). The plates were loaded onto the high-content confocal microscope (Operetta CLS, PerkinElmer) for long term imaging. A 5-minute interval and 200 timepoints in total were used to capture picture of live cells using the digital phase contrast (DPC) mode. Cell tracking properties were analyzed with Harmony 4.9 software (PerkinElmer).

### Live cell mitochondria imaging and analysis

HTR-8/SVneo cells were seeded in a 96-well CellCarrier Ultra plate (6055308, PerkinElmer) at a concentration of 2500 cells per well. 24 hours later, label mitochondria with live cell mito-tracker (C1034, Beyotime) for 10 min at 37 °C following the instructions. Image mitochondria with the Operetta CLS high content confocal system (Operetta CLS, PerkinElmer) immediately after labeling. Photoshop software was used to analysis the mitochondria length and tubule connection.

### Transmission electron microscope (TEM) study

HTR-8/SVneo cells were fixed with Gluta fixation solution (P1126, Solarbio) and collected for transmission electron microscopy (TEM) imaging. Embed the cells with agarose and fix the cells with 1% osmium tetroxide (OsO4) (18456, Ted Pella Inc) in 0.1 M phosphate buffer (PB) (pH 7.4) for 2 h at room temperature. After removing OsO4, the tissues were rinsed with 0.1 M PB (pH 7.4) for 3 times, 15 minutes each. Dehydrate cells with 30%, 50%, 70%, 80%, 95%, and 100% ethanol for 20 minutes each. Then embed cells with aceton (10000418, Sinaopharm Group Chemical Reagent Co. LTD) and EMBed812 (90529-77-4, SPI). Polymerize the embedding models with cells inside and section cells into ultrathin sections, 60-80 nm thin, on the ultra-microtome (Leica UC7, Leica). And the tissues were fished out onto the 150 meshes cuprum grid with formvar film. Staine sections with 2% uranium acetate saturated alcohol solution avoid light for 8 minutes, rinse sections in 70% ethanol for three times and then rinse in ultra-pure water for three times. 2.6% lead citrate avoids CO_2_ staining for 8 minutes, and then rinse with ultra-pure water for 3 times. After being dried by the filer paper, the cuprum grids were put into the grid board and dried overnight at room temperature. The cuprum grids were observed and imaged under TEM (HT7800, Hitachi) and imaging.

### Proteomics and post-translational modification (PTM) analysis

The protein mass spectrum for proteomics and sulhydration of PTM were conducted by the Jingjie PTM BIO company (Hangzhou). In brief, prepare 1gram of total protein from HTR-8/SVneo cells from the control and GYY4137 treated groups, and sent the proteins for quality and quantity tests.

### Whole cell and mitochondria ROS tests

HTR-8/SVneo cells were seeded on a 96-well Cellcarrier ultra plate. After treatments, Mitochondria specific ROS production was detected with MitoSOX Mitochondrial Superoxide Indicators (M36008, ThermoFisher) following the instructions. In brief, add MitoSOX red to culture medium with the final concentration of 1 µM, and incubated cells at 37 °C, with 5% CO_2_ for 30 minutes. Wash the cells three times with warm HBSS (with Calcium and Magnesium). Images were captured with a confocal microscope (Ti2-S-HU, Nikon) and analyzed with Image J software.

### ATP synthesis analysis

The ATP level in HTR-8/SVneo cells was determined following the standard protocol of the ATP content assay kit (S0026, Beyotime). Measure the ATP content of cells in different groups according to the instructions of the reagent kit. The ATP contents (μmol/10^4^ cell) of the samples are calculated by comparison with the standard sample. A total of 3 biological replicates were performed in each group, with 3 technical replicates for one sample.

### Mitochondria membrane potential (Δψm) tests

HTR-8/SVneo cells were seeded into 24-well plates (5 × 10^6^/well per well) and cultured for 48 hours. The JC-1 Kit (S2006, Beyotime) was used according to the manufacturer’s instructions. In brief, cells were stained with JC-1 dye for 10 min at 37 °C, then washed with JC-1 buffer. Further, representative images were obtained using confocal microscopy (Ti2-S-HU, Nikon). JC-1 dye was gathered in the mitochondrial matrix and bright red fluorescence represented high Δψm, while JC-1 monomer was presented in the cytosol and green fluorescence indicated Δψm collapse. The changes in the Δψm were measured by a bright red/green fluorescence ratio.

### Mitochondria oxygen consumption rate detection

To assess mitochondria functions, mitochondria oxygen consumption rate (OCR) was detected with a Seahorse XF Pro analyzer (Agilent). The Seahorse XF cell Mito Stress test kit (103015-100, Agilent), DMEM (103575-100), 200 mM Glutamine solution (103579-100, Agilent), and Seahorse XF Pro M FluxPak (103775-100, Agilent) were used for the HTR-8/SVneo OCR test. Before the experiment, the proper cell counts and FCCP concentration for OCR recording were tested. Then seed HTR-8/SVneo cells (7000/well) with different treatments into the XF 96 well plate for 24 hours before testing. Oligomycin 1.5 µM, FCCP 2.5 µM, and Rotenone & Antimycin A 0.5 µM were used for OCR testing. After seahorse experiment, the total proteins were detected with the BCA kit or staining the cells with 1 µM Hochest33342 for cell counting in each well. The data were recorded and analyzed with Seahorse Wave Pro software. Wells that with drug injection failure were excluded from statistical and graph analysis. Each group got 4 replicates and was normalized with the corresponding cell numbers or total proteins. Figures were plotted with GraphPad Prism8.

### Statistical analysis

The data are presented as the mean ± SEM. Differences between two groups were assessed using the Student’s t-test after normal distribution test. *F* test was used for the variance homogeneity between groups before ANOVA test (GraphPad Prism 8) followed by post-hoc tests (one-way, Tukey; two-way, Bonferroni.) when comparing means of more than two groups. *P* < 0.05 was considered as significant difference.

## Supplementary information

Table S2

Table S3

Supplemental materials

Original WB band

## Data Availability

The data that support the findings of this study are available on request from the corresponding author Yan Li, yli@email.sdfmu.edu.cn.
