# Supplementary material for: Miro2 sulfhydration by CBS/H2S promotes human trophoblast invasion and migration via regulating mitochondria dynamics
Source: Cell Death Dis. 2024 Oct 26;15(10):776. doi: 10.1038/s41419-024-07167-7 (PMC11513031; doi:10.1038/s41419-024-07167-7)
Supplement: Supplementary file 3 — Supplemental materials [file 41419_2024_7167_MOESM3_ESM.docx]

**Miro2 Sulfhydration by CBS/H_2_S promotes human trophoblast invasion and migration via regulating mitochondria dynamics**

Hao Feng^1 §^, Zongxin Sun^1, 2 §^, Baoshi Han^3^, Huitang Xia^4^, Lumei Chen^1^, Chunlei Tian^1^, Suhua Yan^5^, Yugen Shi^5^, Jie Yin^4^, Wengang Song^6^, Peipei Gong^7^, Shuanglian Wang^8 *^, Yan Li^6, 9 *^

^1^ Department of Obstetrics & Gynecology, the First Affiliated Hospital of Shandong First Medical University & Shandong Provincial Qianfoshan Hospital, Jinan, 250014, China

^2^ Department of emergency, Affiliated Hospital of Chifeng University, Shifeng, 024001, China

^3^ Institute of Women, Children and Reproductive Health, Shandong University, Jinan, 250012, China

^4^ Jinan Clinical Research Center for Tissue Engineering Skin Regeneration and Wound Repair，Jinan, Shandong, 250014, P. R. China.

^5^ Department of Cardiology, the First Affiliated Hospital of Shandong First Medical University & Shandong Provincial Qianfoshan Hospital, Jinan, 250014, China

^6^ Shandong Provincial Key Laboratory for Rheumatic Disease and Translational Medicine, The First Affiliated Hospital of Shandong First Medical University & Shandong Provincial Qianfoshan Hospital, Jinan, 250012, China.

^7^ Department of Rehabilitation Medicine, the Provincial Hospital Affiliated to Shandong First Medical University, Jinan, 250021, China

^8^ Medical Science and Technology Innovation Center, Shandong First Medical University & Shandong Academy of Medical Sciences, Jinan, 250012, China

^9^ Translational Medical Research Centre, the First Affiliated Hospital of Shandong First Medical University & Shandong Provincial Qianfoshan Hospital, Jinan, 250014, China

^§^ These authors contributed equally to this work.

^*^To whom correspondence should be addressed. Yan Li, e-mail address: yli@email.sdfmu.edu.cn; Shuanglian Wang, email address: [wsl6319@sdu.edu.cn](mailto:wsl6319@sdu.edu.cn)

Supplemental Figures


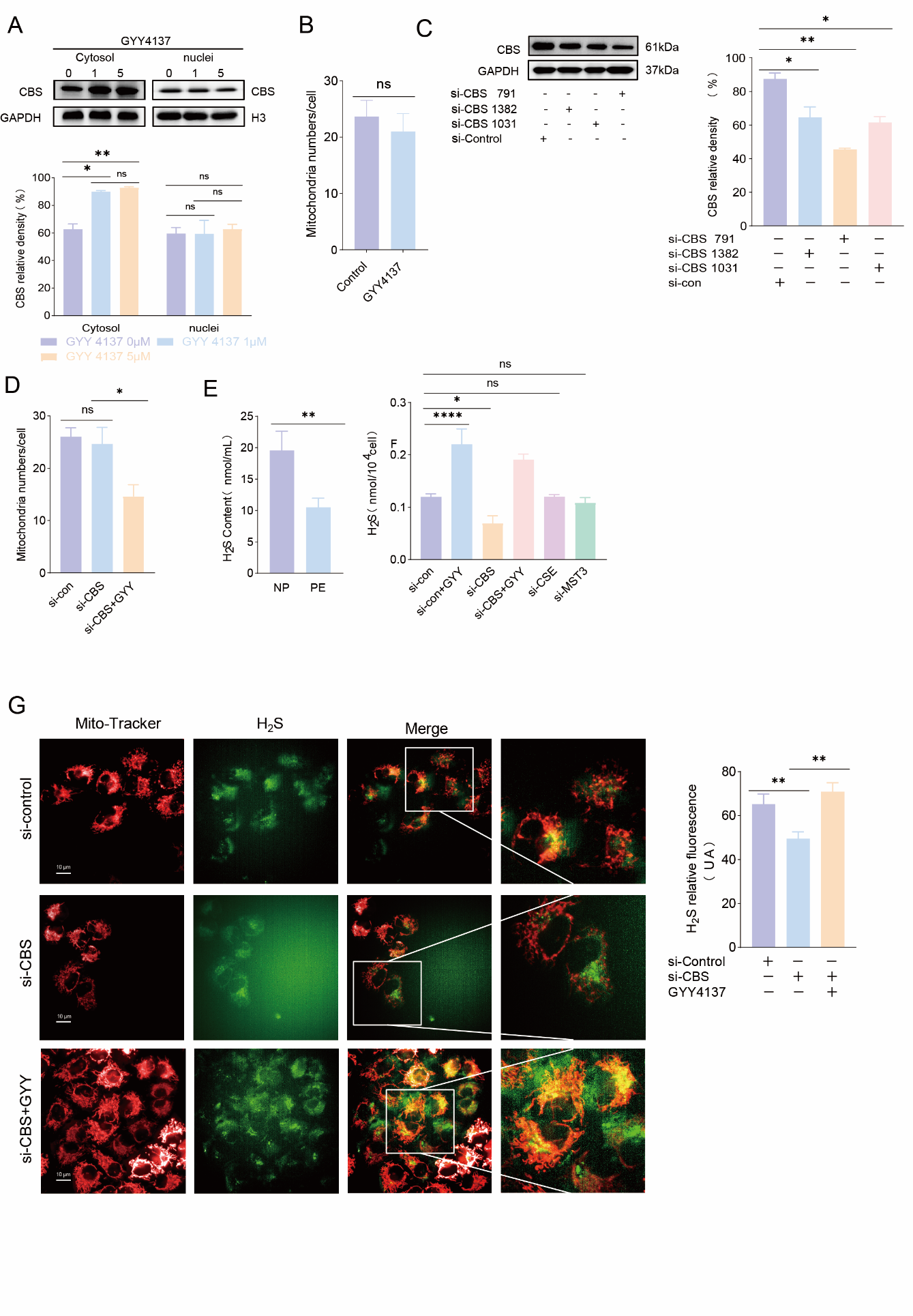


Supplemental Figure 1

A, CBS protein detection on cytosol and nuclei with and without GYY4137 stimuli using western blot. Statistical bar graph showed the relative CBS expression between groups. B, Statistical bar graph analyzed the total mitochondria numbers per cell with and without GYY4137 (1 µM) treatment. n=6 each group. C, Western blot method detected the efficiency of CBS siRNAs. Statistical bar graph showed CBS protein differences between groups. n=6 each group. D, Statistical bar graph analyzed the total mitochondria numbers per cell in si-Control, si-CBS and si-CBS+GYY4137 groups. n=6 each group E, Bar graph of H_2_S content in PE and NP patents plasma. n=10 each group. F, Bar graph of H_2_S content in HTR-8/SVneo cells in si--Control, si-Control +GYY4137, si-CBS, si-CBS+GYY4137, si-CSE, si-MST3 groups. n=6 each group. G, Double labeling of mitochondria and H_2_S with Mito-Tracker (red) and H_2_S probe (green) in si-Control, si-CBS and si-CBS+GYY4137 groups. Bar graph showed the statistical difference between the three groups. n=6 each group. NP: Normal pregnancy; PE: Preeclampsia. ‘ns’: no significance. ‘*’: *P*< 0.05; ‘**’: *P*< 0.01; ‘****’: *P*<0.0001.


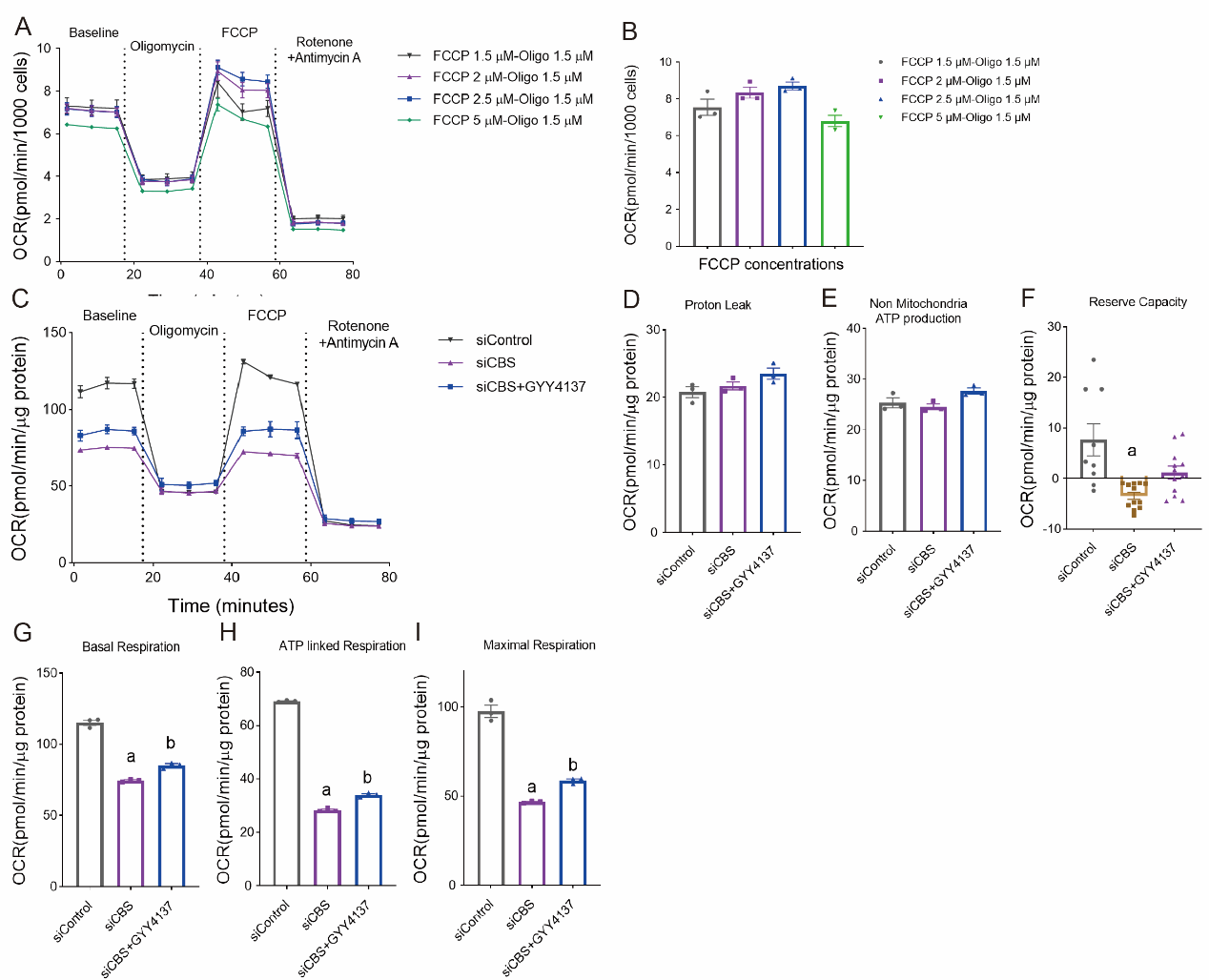


Supplemental Figure2

A, Oxygen consumption rate (OCR) of HTR-8/SVneo cells in response to Oligomycin, different FCCP (1.5 µM, 2 µM, 2.5 µM, and 5 µM) concentrations, and Rotenone+Antimycin A. B, Bar graph of OCR under to different FCCP concentrations to find the proper FCCP concentration. C, OCR trace in si-Control, si-CBS, si-CBS+GYY4137 groups in response to to Oligomycin (1.5 µM), FCCP concentrations (2.5 µM), and Rotenone+Antimycin A (0.5 µM). D-F, Bar graph of basal respiration, ATP linked respiration, and Maximal respiration in si-Control, si-CBS, si-CBS+GYY4137 groups. ‘a’: *P*<0.05 comparing with si-Control; ‘b’: *P*<0.05 comparing with si-CBS+GYY4137 group. G-I, Bar graph of Proton leak, Non mitochondria ATP production, and Reserve capacity in si-Control, si-CBS, si-CBS+GYY4137 groups. ‘a’: *P*<0.05 comparing with si-Control. n=7 each group.


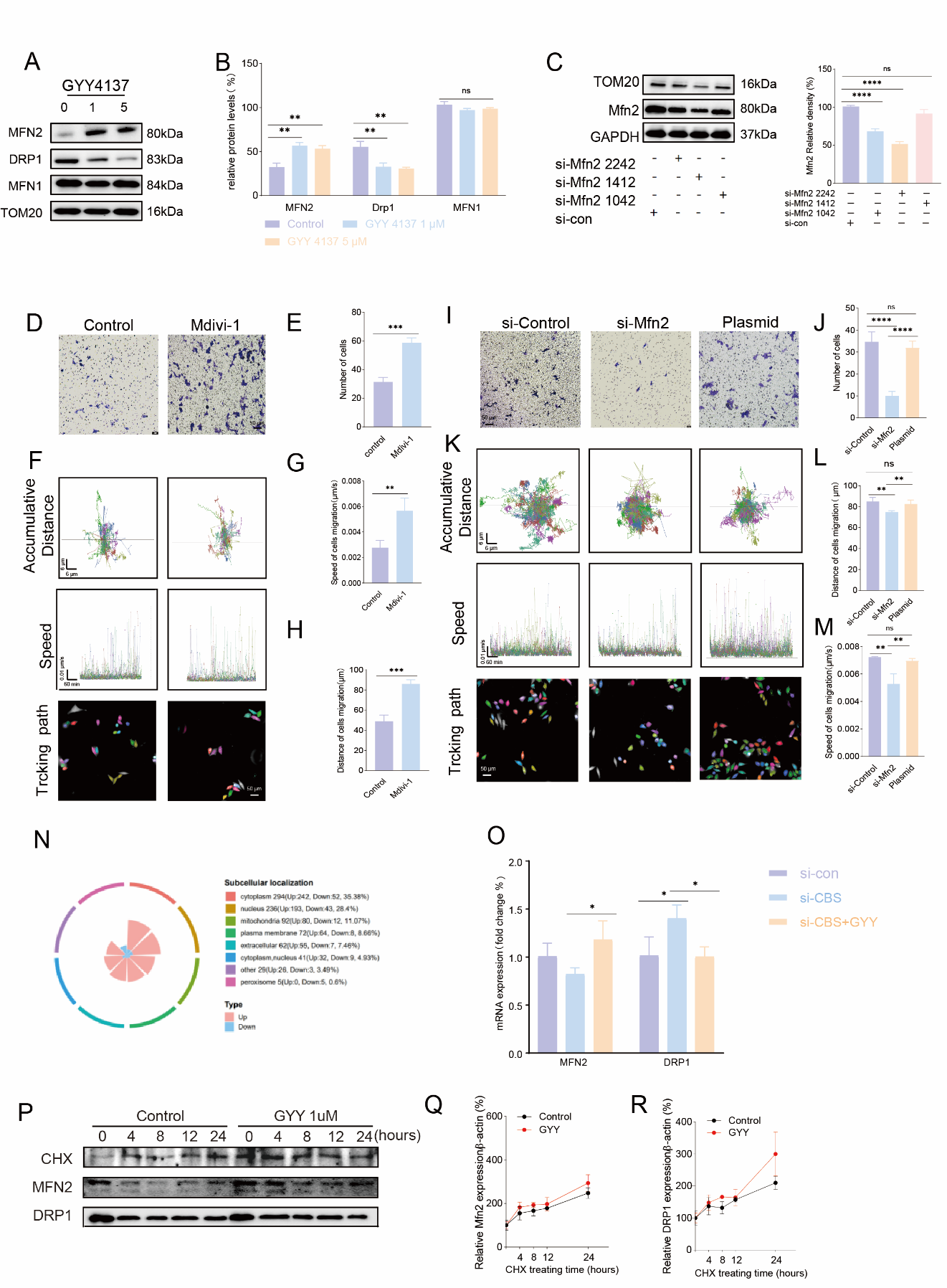


Supplemental Figure3

A-B, Western blot method tested mitochondria dynamic related proteins (MFN1 and 2, Drp1) expression in response to low dose GYY4137 (1 µM and 5 µM). Statistical bar graph showed the differences between groups. n=7 each group. C-D, Transwell study showed the penetrated HTR-8/SVneo cell numbers in Control and Mdivi-1 group. Statistical bar graph showed increased cell penetration through transwell comparing with Control group. n=7 each group. E-G, Live-cell tracking distance and speed were recorded and analyzed with confocal microscope. The accumulative distance and moving speed were increased in Mdivi-1 group. n=7 each group. H, Western blot method detected the the efficiency of Mfn2 siRNAs. Statistical bar graph showed Mfn2 protein differences between groups. n=6 each group. I and J, Transwell study showed the penetrated HTR-8/SVneo cell numbers in si-Control and si-Mfn2 group. Statistical bar graph showed decreased cell penetration through transwell comparing with si-Control group. n=7 each group. K-M, Live-cell tracking distance and speed were recorded and analyzed with confocal microscope. The accumulative distance and moving speed were decreased in si-Mfn2 group. N, Subcellular distribution of the differentially sulfhydrated proteins enrichment in HTR-8/SVneo. O, qPCR results showed the relative mRNA levels of MFN2/DRP1 in si-Con, si-CBS and si-CBS+GYY groups. n=3 in each group. P-R, Western blot bands showed the protein levels of MFN2 and Drp1 in response to CHX (200µg/ml) with different incubation time (0, 4, 8, 12, and 24 hours). n=3 for each group. ‘ns’: no significance. ‘*’: *P*< 0.05; ‘**’: *P*< 0.01; ‘***’: *P*<0.001; ‘****’: *P*<0.0001.


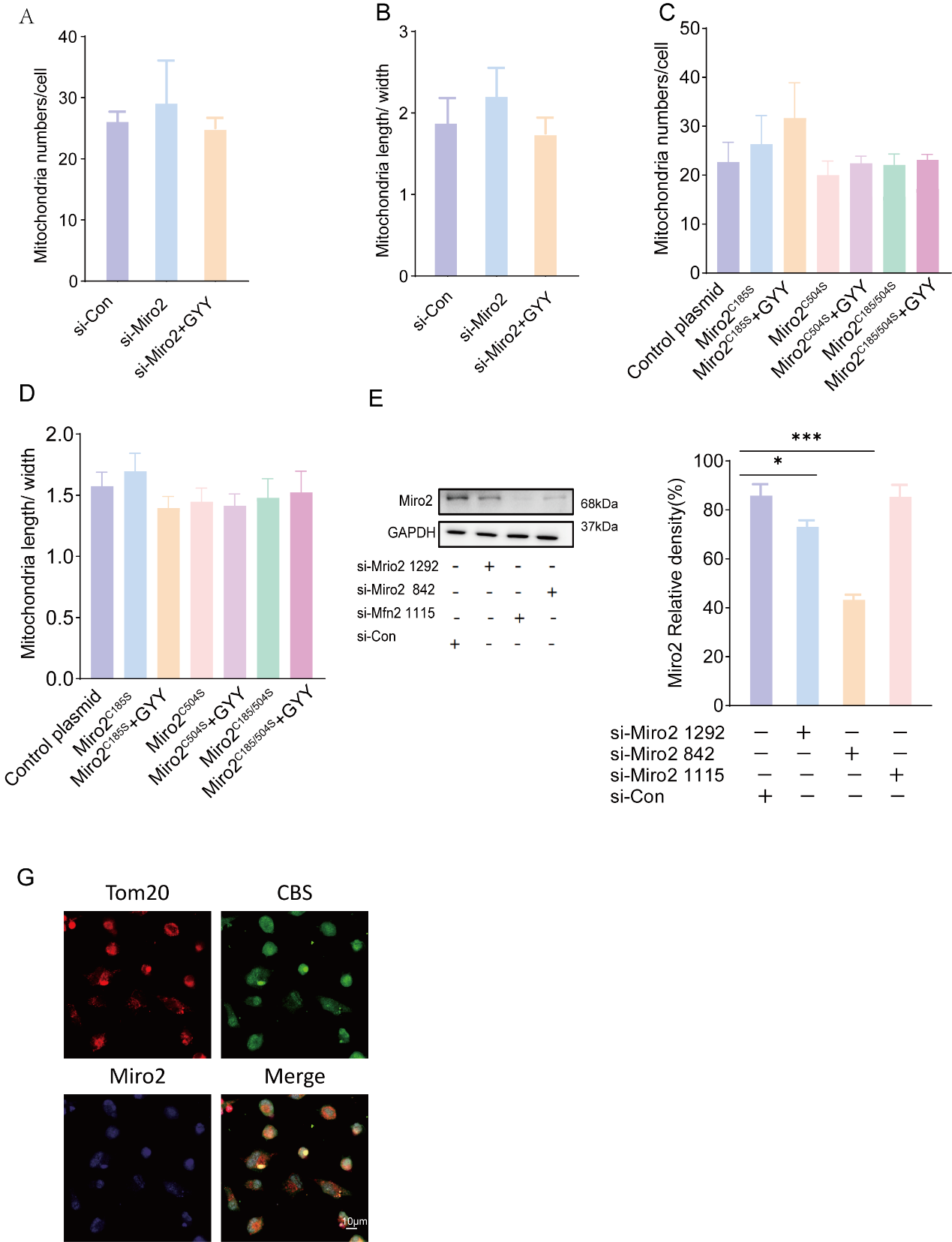


Supplemental Figure4

A-B, Statistical bar graph showed the mitochondria numbers per cell and length/width in si-Control, si-Miro2 and si-Miro2+GYY4137 groups. n=6 each group. C-D, Statistical bar graph showed the mitochondria numbers per cell and length/width in Control plasmid, Miro2^C185S^, Miro2^C504S^, Miro2^C185S/C504S^ with or without GYY4137 groups. n=6 each group. E, Western blot method detected the knockdown efficiency of Miro2 siRNAs. Statistical bar graph showed CBS protein differences between groups. n=6 each group. F, Fluorescent staining of Tom20 (red, to mark mitochondria), CBS (green) and Miro2 (purple) in HTR-8/SVneo. ‘ns’: no significance. ‘*’: *P*< 0.05; ‘***’: *P*<0.001.


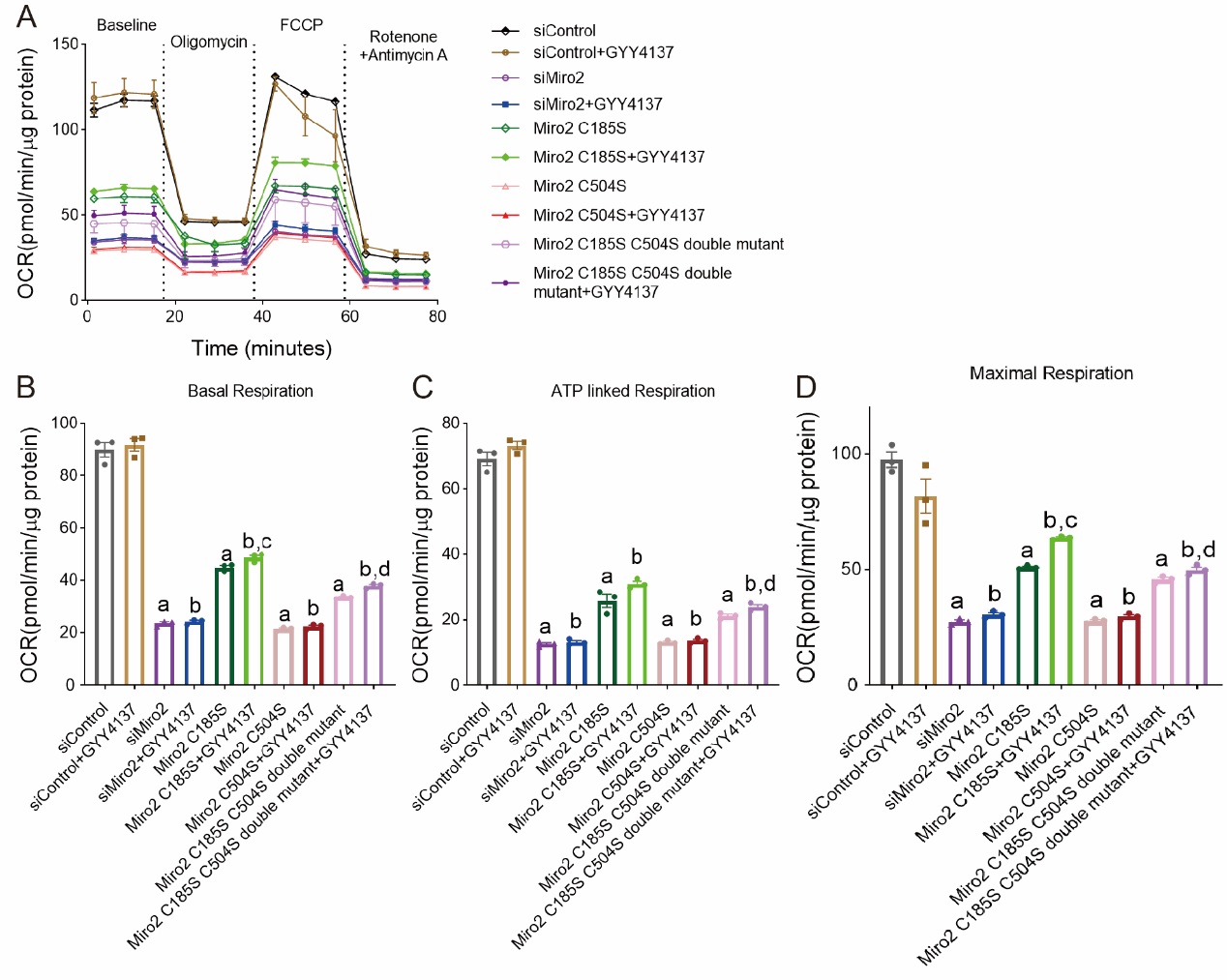


Supplemental Figure5

A, OCR trace of HTR-8/SVneo cells in Control plasmid, si-Miro2, Miro2^C185S^, Miro2^C504S^, Miro2^C185S/C504S^ with or without GYY4137 groups. B-D, Bar graph of basal respiration, ATP linked respiration, and Maximal respiration in different groups. ‘a’: *P*<0.05 comparing with Control plasmid; ‘b’: *P*<0.05 comparing with Control plasmid+GYY4137 group; ‘c’: *P*<0.05 comparing with Miro2^C185S^; ‘d’: *P*<0.05 comparing with Miro2^C504S^; ‘e’: *P*<0.05 comparing with Miro2^C185S^+GYY4137; ‘f’: *P*<0.05 comparing with Miro2^C504S^+GYY4137. n=7 each group.


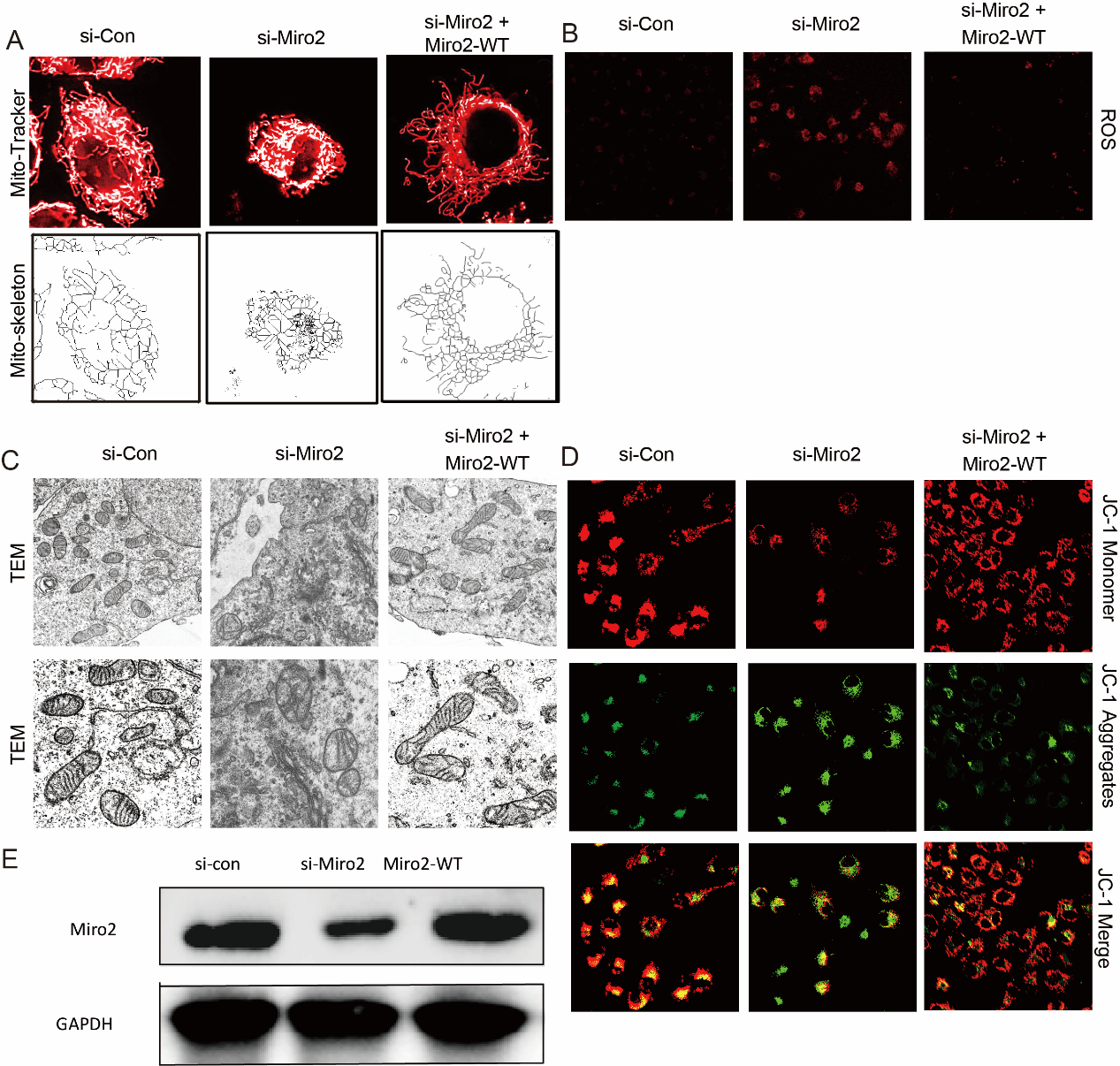


Supplemental Figure6

The rescue experiments were performed with applying Miro2-WT plasmid to si-Miro group to assess if the efficiency of si-Miro2. A, Live-cell mitochondria tracking in si-Control plasmid, si-Miro2 and si-Miro2+Miro2-WT. B, ROS detector showed similar fluorescence in si-Contorl and si-Miro2+Miro2-WT plasmid groups. C, TEM images showed relative normal mitochondria size, shapes and cristae in si-Contorl and si-Miro2+Miro2-WT plasmid groups. D, Δψm was assessed using JC-1 fluorescent detector (red in aggregates, green in monomer). Δψm was lower in si-Miro2 group, while si-Contorl and si-Miro2+Miro2-WT plasmid groups showed similar green and red fluorescence.

| Supplemental Table 1 PCR primers and siRNA sequences | |
| --- | --- |
| Primer name | Sequence（5’to3’） |
| hCBS-F | TGAGATTGTGAGGACGCCCAC |
| hCBS-R | TCACACTGCTGCAGGATCTC |
| hL19-F | AGACCCCAATGAGACCAATG |
| hL19-R | GTGTTTTTCCGGCATCGAGC |
| hDrp1-F | CAAAGCAGTTTGCCTGTGGA |
| hDrp1-R | TCTTGGAGGACTATGGCAGC |
| hMFN2-F | TGCAGGTGTAAGGGACGATT |
| hMFN2-F | GAGGCTCTGCAAATGGGATG |
| si-RNA name | Sequence（5’to3’） |
| Control siRNA | GUAUGACAACAGCCUCAAGTT |
| CBS（human）siRNA- 1031 | CCAGGAAGCUGAAGGAGAATT |
| CBS（human）siRNA- 1382 | UGACCAAGUUCCUGAGCGATT |
| CBS（human）siRNA-791 | GGGCUGAGAUUGUGAGGACTT |
| Miro2（human）siRNA- 1292 | UCAAUGGACAGGAGAAGUATT |
| Miro2（human）siRNA- 842 | AGAGAGUGUUUGAGAAGCATT |
| Miro2（human）siRNA-1115 | UGGUCCAGCCUCUUCUCACTT |
| MFN2（human）siRNA-2264 | GGUUGGACAGUGAGCUCAATT |
| MFN2（human）siRNA-1046 | GGAUGUUUGAGUUUCAGAATT |
| MFN2（human）siRNA-1412 | CAGUAGUCCUCAAGGUUUATT |
| Control plasmid | CTTGGTACCGAGCTCGGATCCGCCACC |
| hCSE siRNA-1 | GCCCAGUUCCUGGAAUCUAAUdTdT |
| hCSE siRNA-2 | CCAAGCUUGUUUGGAUCGAAAdTdT |
| hCSE siRNA-3 | CGUCAGUGUACAGGUUGUACAdTdT |
| hMST siRNA-1 | CACGCCGUGUCACUGCUUGAUdTdT |
| hMST siRNA-2 | UGAGGAGAUCCGCCAUCUGUUdTdT |
| hMST siRNA-3 | GAAGAAAGUGGACCUGUCUAAdTdT |

Supplemental table2 and 3 (Table S2, Table S3) are supplied separately.

Supplemental Table 4

Miro2 [NM_138769.3](https://www.ncbi.nlm.nih.gov/nuccore/NM_138769.3)  sequence

atgaggcgggacgtgcgcatcctgttactgggcgaggcccaggtggggaagacgtcgctgatcctgtccctggtgggcgaggagttccccgaggaggtccctccccgcgcggaggagatcacGatccccgcggacgtcaccccggagaaggtgcccacccacatcgtggactactcagaagccgagcagacggacgaggagctgcgggaggagatccacaaggcaaacgtggtgtgtgtggtgtatgacgtctctgaggaggccaccattgagaagattcgaactaagtggatcccactggtgaatggggggaccacgcaggggcccagggtgcccatcatcctagtgggcaacaagtcagacctgcggtcggggagctccatggaggccgtgctccccatcatgagccagtttcccgagattgagacctgcgtggagtgttcggccaagaacctgaggaacatctcagagctgttctactacgcccagaaggccgtcctgcatcccacagcccccctctatgaccctgaggccaagcagttgaggcccgcgtgcgcccaggcgctgacgcgcatcttcaggctctcagatcaggacctggaccaggcgctcagtgacgaagagctcaacgctttccagaaatcctgctttgggcaccccctggccccgcaggccctggaggacgtgaagacggtggtgtgcaggaacgtggcgggcggcgtgcgggaggaccggctgaccctggatggtttcctcttcctgaacacgctcttcatccagcgcggccggcacgagaccacctggaccatcctgcggcgcttcggctacagcgatgccctggagctgactgcggactatctctcccctctgatccacgtgccccccggctgcagcacggagctcaaccaccttggctaccagtttgtgcagagagtgtttgagaagcacgaccaggaccgcgacggcgccctctcgcccgtggagctgcaaagccttttcagtgtgttcccagcagcgccctggggccccgagctcccacgcacagtccgcacagaggccggccggttgcccctgcacggatacctctgccagtggaccctggtgacctacctggacgtccggagctgccttggacacctaggctacctg

ggctaccccaccctctgtgagcaggaccaggcccatgccatcacagtcactcgtgagaagaggctggaccaggagaaggg

acagacgcagcggagcgtcctcctgtgcaaggtggtaggggcccgtggagtgggcaagtctgccttcctgcaggcTtttc

tcggccgcggcctggggcaccaggacacgagggagcagcctcccggctacgccatcgacacggtgcaggtcaatggacag

gagaagtacttgatcctctgtgaggtgggcacagatggtctgctggccacatcgctggacgccacctgtgacgttgcctg

cttgatgtttgatggcagtgacccaaagtcctttgcacattgtgccagcgtctacaagcaccattacatggacgggcaga

ccccctgcctctttgtctcctccaaggccgacctgcccgaaggtgtcgcggtgtctggcccatcaccggccgagttttgc

cgcaagcaccggctacccgctcccgtgccgttctcctgtgctggcccagccgagcccagcaccaccatcttcacccagct

cgccaccatggccgccttcccacatttggtccacgcagagctgcatccctcttccttctggctccgggggctgctggggg

ttgtcggggccgccgtggccgcagtcctcagcttctcactctacagggtcctggtgaagagccagtga

Miro2 C185S

atgaggcgggacgtgcgcatcctgttact

gggcgaggcccaggtggggaagacgtcgctgatcctgtccctggtgggcgaggagttccccgaggaggtccctccccgcg

cggaggagatcacGatccccgcggacgtcaccccggagaaggtgcccacccacatcgtggactactcagaagccgagcag

acggacgaggagctgcgggaggagatccacaaggcaaacgtggtgtgtgtggtgtatgacgtctctgaggaggccaccat

tgagaagattcgaactaagtggatcccactggtgaatggggggaccacgcaggggcccagggtgcccatcatcctagtgg

gcaacaagtcagacctgcggtcggggagctccatggaggccgtgctccccatcatgagccagtttcccgagattgagacc

tgcgtggagtgttcggccaagaacctgaggaacatctcagagctgttctactacgcccagaaggccgtcctgcatcccac

agcccccctctatgaccctgaggccaagcagttgaggcccgcgTCTgcccaggcgctgacgcgcatcttcaggctctcag

atcaggacctggaccaggcgctcagtgacgaagagctcaacgctttccagaaatcctgctttgggcaccccctggccccg

caggccctggaggacgtgaagacggtggtgtgcaggaacgtggcgggcggcgtgcgggaggaccggctgaccctggatggtttcctcttcctgaacacgctcttcatccagcgcggccggcacgagaccacctggaccatcctgcggcgcttcggctaca

gcgatgccctggagctgactgcggactatctctcccctctgatccacgtgccccccggctgcagcacggagctcaaccac

cttggctaccagtttgtgcagagagtgtttgagaagcacgaccaggaccgcgacggcgccctctcgcccgtggagctgca

aagccttttcagtgtgttcccagcagcgccctggggccccgagctcccacgcacagtccgcacagaggccggccggttgc

ccctgcacggatacctctgccagtggaccctggtgacctacctggacgtccggagctgccttggacacctaggctacctg

ggctaccccaccctctgtgagcaggaccaggcccatgccatcacagtcactcgtgagaagaggctggaccaggagaaggg

acagacgcagcggagcgtcctcctgtgcaaggtggtaggggcccgtggagtgggcaagtctgccttcctgcaggcTtttc

tcggccgcggcctggggcaccaggacacgagggagcagcctcccggctacgccatcgacacggtgcaggtcaatggacag

gagaagtacttgatcctctgtgaggtgggcacagatggtctgctggccacatcgctggacgccacctgtgacgttgcctg

cttgatgtttgatggcagtgacccaaagtcctttgcacattgtgccagcgtctacaagcaccattacatggacgggcaga

ccccctgcctctttgtctcctccaaggccgacctgcccgaaggtgtcgcggtgtctggcccatcaccggccgagttttgc

cgcaagcaccggctacccgctcccgtgccgttctcctgtgctggcccagccgagcccagcaccaccatcttcacccagct

cgccaccatggccgccttcccacatttggtccacgcagagctgcatccctcttccttctggctccgggggctgctggggg

ttgtcggggccgccgtggccgcagtcctcagcttctcactctacagggtcctggtgaagagccagtga

Miro2 C504S

atgaggcgggacgtgcgcatcctgttact

gggcgaggcccaggtggggaagacgtcgctgatcctgtccctggtgggcgaggagttccccgaggaggtccctccccgcg

cggaggagatcacGatccccgcggacgtcaccccggagaaggtgcccacccacatcgtggactactcagaagccgagcag

acggacgaggagctgcgggaggagatccacaaggcaaacgtggtgtgtgtggtgtatgacgtctctgaggaggccaccat

tgagaagattcgaactaagtggatcccactggtgaatggggggaccacgcaggggcccagggtgcccatcatcctagtgg

gcaacaagtcagacctgcggtcggggagctccatggaggccgtgctccccatcatgagccagtttcccgagattgagacc

tgcgtggagtgttcggccaagaacctgaggaacatctcagagctgttctactacgcccagaaggccgtcctgcatcccac

agcccccctctatgaccctgaggccaagcagttgaggcccgcgtgcgcccaggcgctgacgcgcatcttcaggctctcag

atcaggacctggaccaggcgctcagtgacgaagagctcaacgctttccagaaatcctgctttgggcaccccctggccccg

caggccctggaggacgtgaagacggtggtgtgcaggaacgtggcgggcggcgtgcgggaggaccggctgaccctggatggtttcctcttcctgaacacgctcttcatccagcgcggccggcacgagaccacctggaccatcctgcggcgcttcggctaca

gcgatgccctggagctgactgcggactatctctcccctctgatccacgtgccccccggctgcagcacggagctcaaccac

cttggctaccagtttgtgcagagagtgtttgagaagcacgaccaggaccgcgacggcgccctctcgcccgtggagctgca

aagccttttcagtgtgttcccagcagcgccctggggccccgagctcccacgcacagtccgcacagaggccggccggttgc

ccctgcacggatacctctgccagtggaccctggtgacctacctggacgtccggagctgccttggacacctaggctacctg

ggctaccccaccctctgtgagcaggaccaggcccatgccatcacagtcactcgtgagaagaggctggaccaggagaaggg

acagacgcagcggagcgtcctcctgtgcaaggtggtaggggcccgtggagtgggcaagtctgccttcctgcaggcTtttc

tcggccgcggcctggggcaccaggacacgagggagcagcctcccggctacgccatcgacacggtgcaggtcaatggacag

gagaagtacttgatcctctgtgaggtgggcacagatggtctgctggccacatcgctggacgccacctgtgacgttgcctg

cttgatgtttgatggcagtgacccaaagtcctttgcacatTCTgccagcgtctacaagcaccattacatggacgggcaga

ccccctgcctctttgtctcctccaaggccgacctgcccgaaggtgtcgcggtgtctggcccatcaccggccgagttttgc

cgcaagcaccggctacccgctcccgtgccgttctcctgtgctggcccagccgagcccagcaccaccatcttcacccagct

cgccaccatggccgccttcccacatttggtccacgcagagctgcatccctcttccttctggctccgggggctgctggggg

ttgtcggggccgccgtggccgcagtcctcagcttctcactctacagggtcctggtgaagagccagtga

Miro2 C185S C504S

atgaggcgggacgtgcgcatcctgttact

gggcgaggcccaggtggggaagacgtcgctgatcctgtccctggtgggcgaggagttccccgaggaggtccctccccgcg

cggaggagatcacGatccccgcggacgtcaccccggagaaggtgcccacccacatcgtggactactcagaagccgagcag

acggacgaggagctgcgggaggagatccacaaggcaaacgtggtgtgtgtggtgtatgacgtctctgaggaggccaccat

tgagaagattcgaactaagtggatcccactggtgaatggggggaccacgcaggggcccagggtgcccatcatcctagtgg

gcaacaagtcagacctgcggtcggggagctccatggaggccgtgctccccatcatgagccagtttcccgagattgagacc

tgcgtggagtgttcggccaagaacctgaggaacatctcagagctgttctactacgcccagaaggccgtcctgcatcccac

agcccccctctatgaccctgaggccaagcagttgaggcccgcgTCTgcccaggcgctgacgcgcatcttcaggctctcag

atcaggacctggaccaggcgctcagtgacgaagagctcaacgctttccagaaatcctgctttgggcaccccctggccccg

caggccctggaggacgtgaagacggtggtgtgcaggaacgtggcgggcggcgtgcgggaggaccggctgaccctggatggtttcctcttcctgaacacgctcttcatccagcgcggccggcacgagaccacctggaccatcctgcggcgcttcggctaca

gcgatgccctggagctgactgcggactatctctcccctctgatccacgtgccccccggctgcagcacggagctcaaccac

cttggctaccagtttgtgcagagagtgtttgagaagcacgaccaggaccgcgacggcgccctctcgcccgtggagctgca

aagccttttcagtgtgttcccagcagcgccctggggccccgagctcccacgcacagtccgcacagaggccggccggttgc

ccctgcacggatacctctgccagtggaccctggtgacctacctggacgtccggagctgccttggacacctaggctacctg

ggctaccccaccctctgtgagcaggaccaggcccatgccatcacagtcactcgtgagaagaggctggaccaggagaaggg

acagacgcagcggagcgtcctcctgtgcaaggtggtaggggcccgtggagtgggcaagtctgccttcctgcaggcTtttc

tcggccgcggcctggggcaccaggacacgagggagcagcctcccggctacgccatcgacacggtgcaggtcaatggacag

gagaagtacttgatcctctgtgaggtgggcacagatggtctgctggccacatcgctggacgccacctgtgacgttgcctg

cttgatgtttgatggcagtgacccaaagtcctttgcacatTCTgccagcgtctacaagcaccattacatggacgggcaga

ccccctgcctctttgtctcctccaaggccgacctgcccgaaggtgtcgcggtgtctggcccatcaccggccgagttttgc

cgcaagcaccggctacccgctcccgtgccgttctcctgtgctggcccagccgagcccagcaccaccatcttcacccagct

cgccaccatggccgccttcccacatttggtccacgcagagctgcatccctcttccttctggctccgggggctgctggggg

ttgtcggggccgccgtggccgcagtcctcagcttctcactctacagggtcctggtgaagagccagtga
