# Supplementary material for: Miro2 sulfhydration by CBS/H2S promotes human trophoblast invasion and migration via regulating mitochondria dynamics
Source: Cell Death Dis. 2024 Oct 26;15(10):776. doi: 10.1038/s41419-024-07167-7 (PMC11513031; doi:10.1038/s41419-024-07167-7)

Figure1-B

MST-3

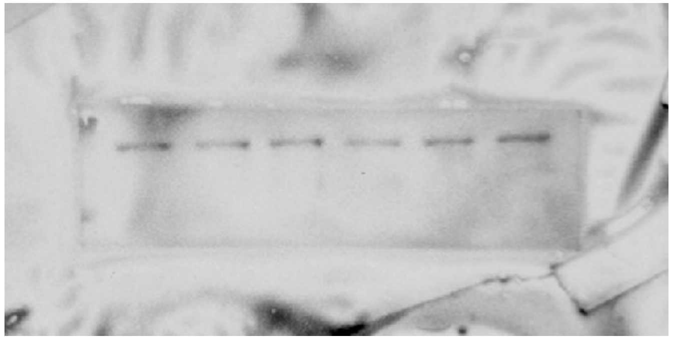

CBS

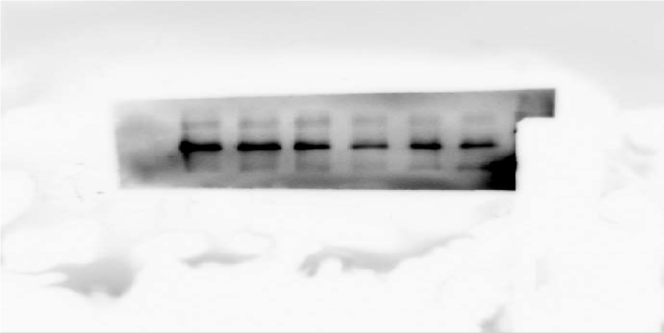

CSE-

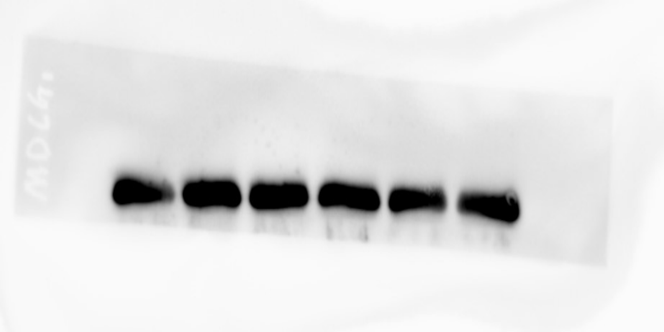

GAPDH-

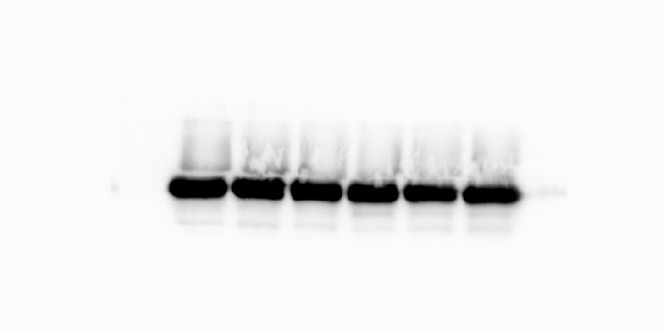

Figure2-A

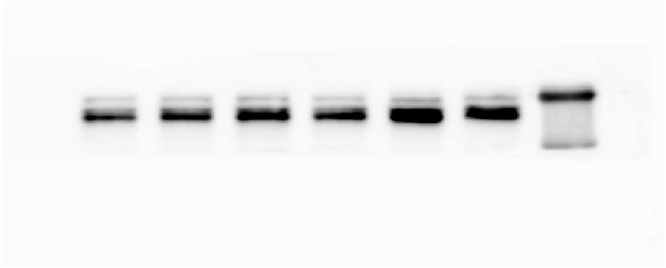

CBS

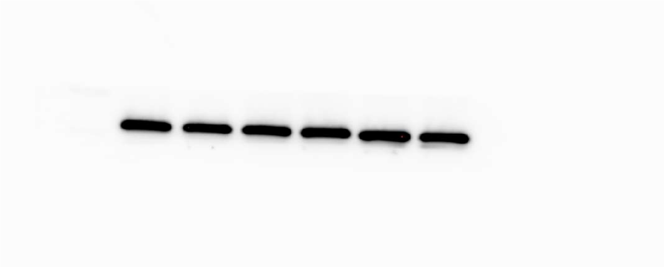

CSE

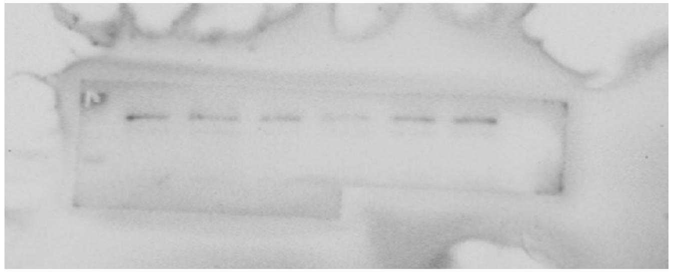

MST3

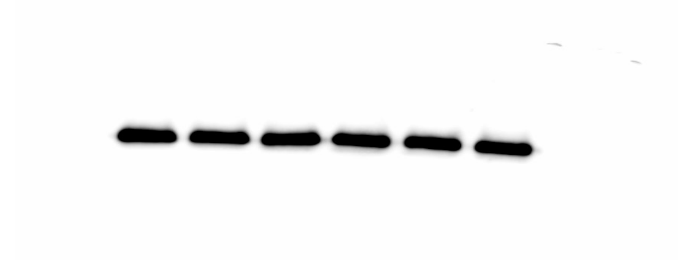

GAPDH

Figure2-C

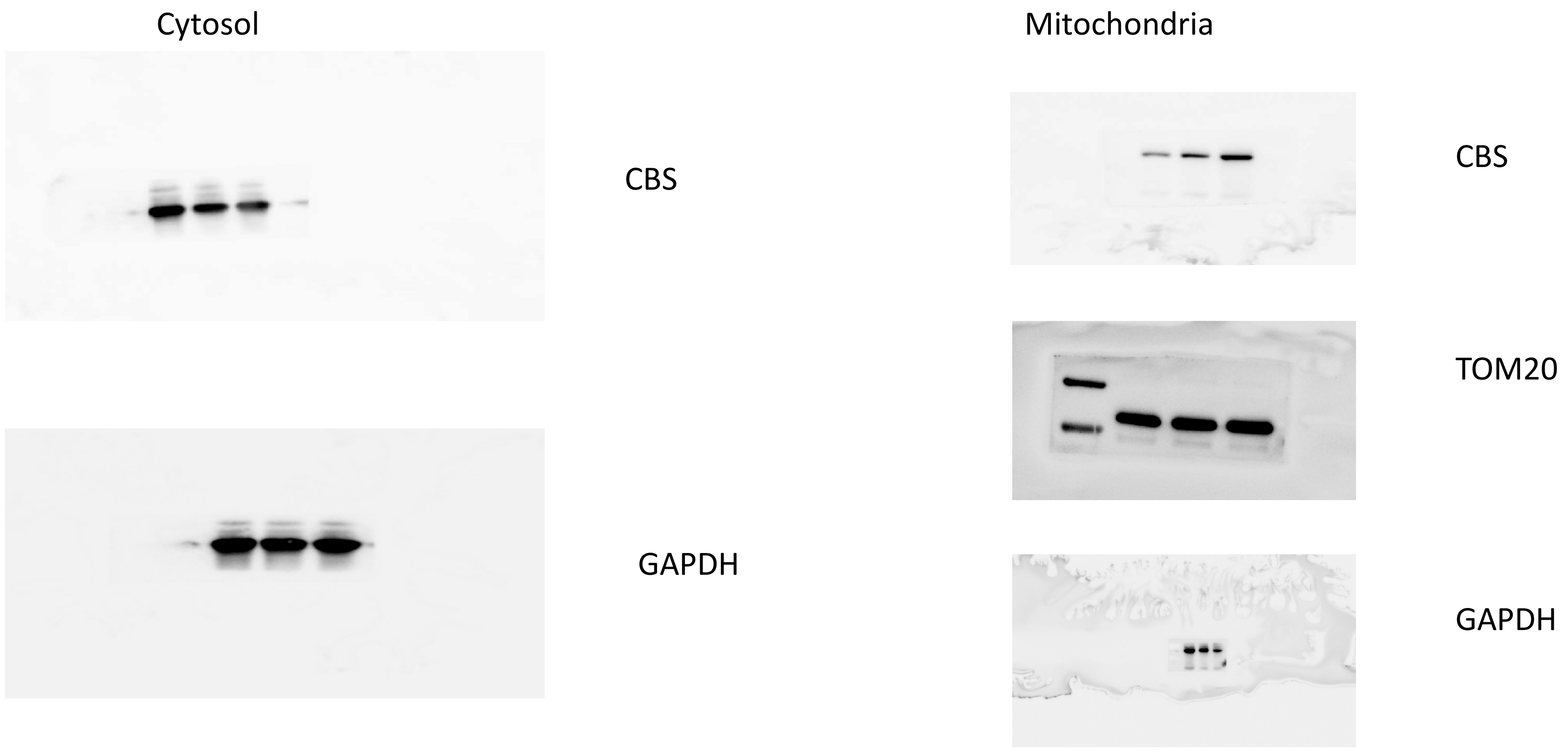

Figure2-E

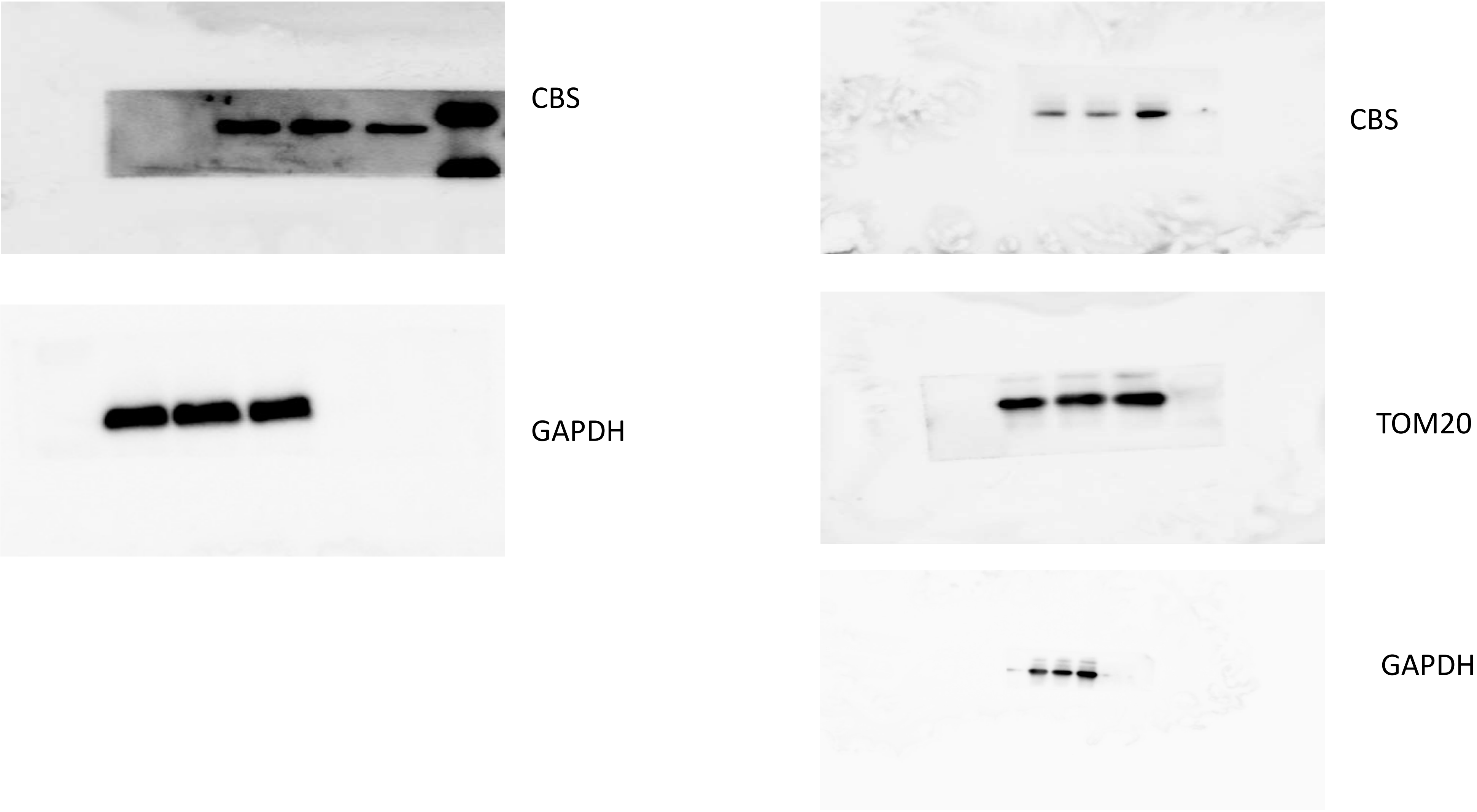

Figure2-H

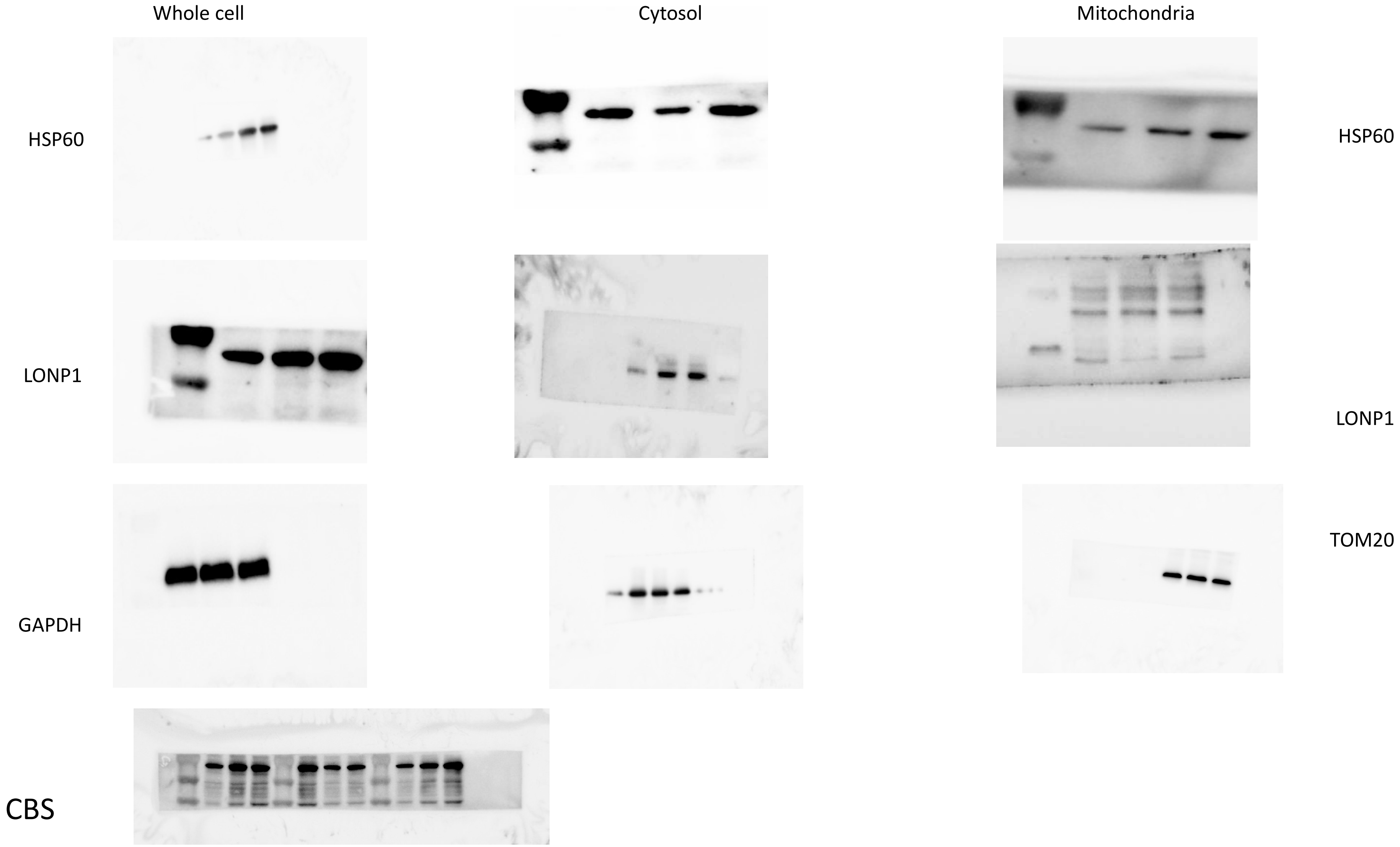

Whole cell
Cytosol
Mitochondria
HSP60
HSP60
LONP1
LONP1
TOM20
GAPDH
CBS

Figure5-G

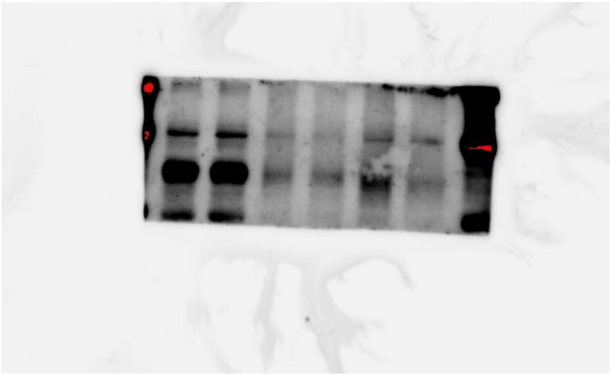

MFN2

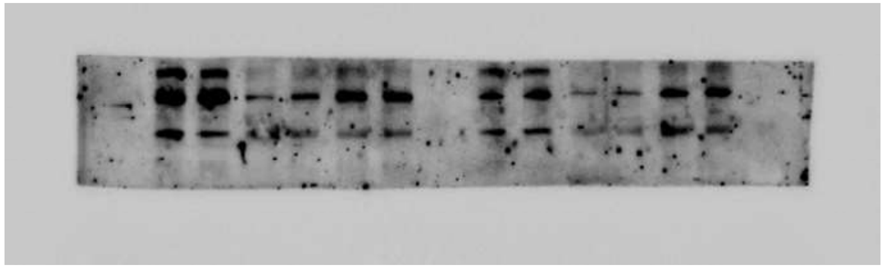

DRP1

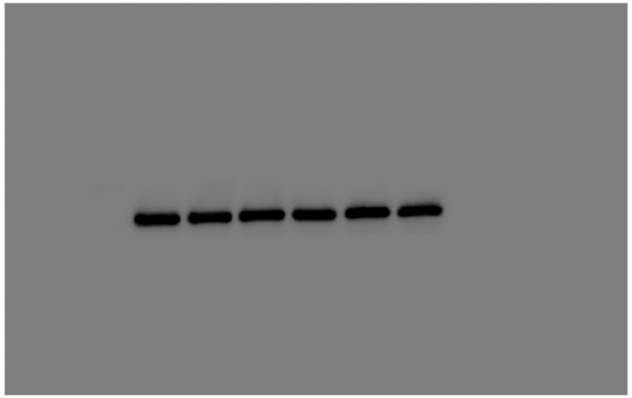

GAPDH

Figure6-G

Sulfhydrated Miro2

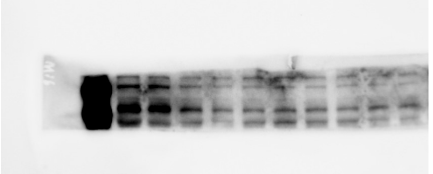

Total Miro2

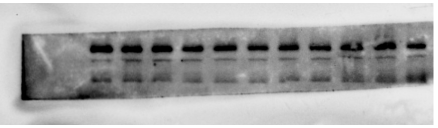

GAPDH

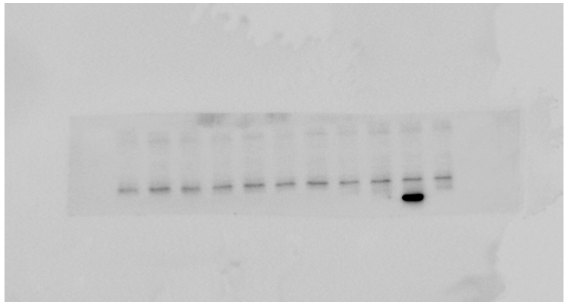

Figure6-F

Miro2

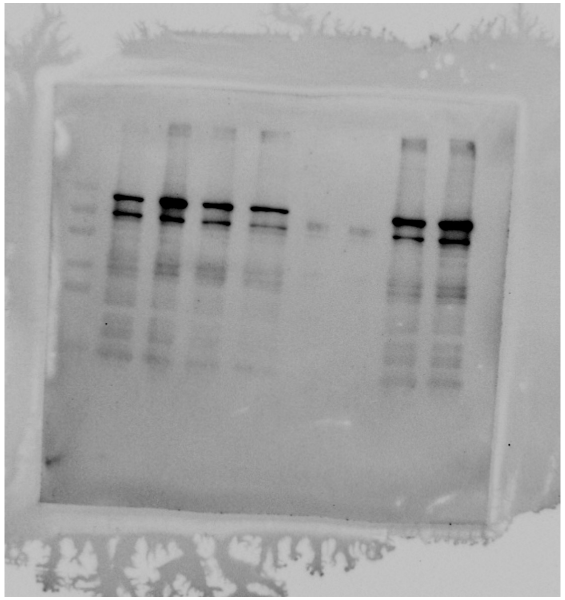

Supplementary Figure 1A

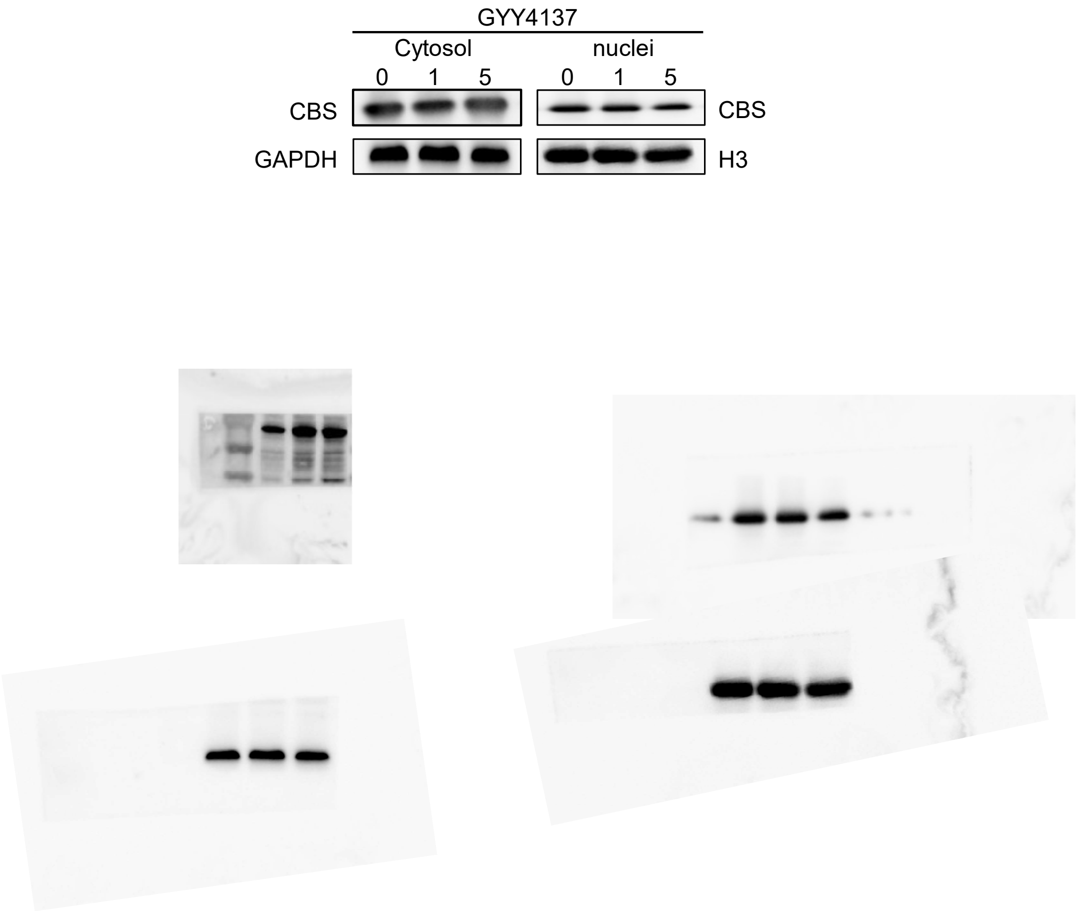

Supplementary Figure 1C

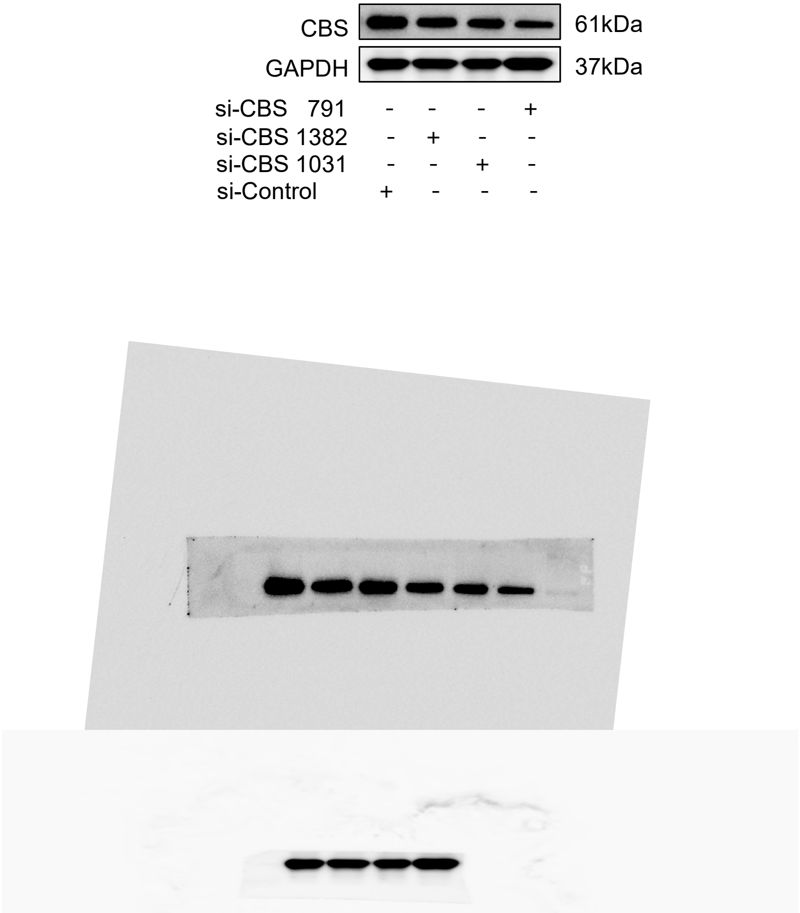

Supplementary Figure 3A

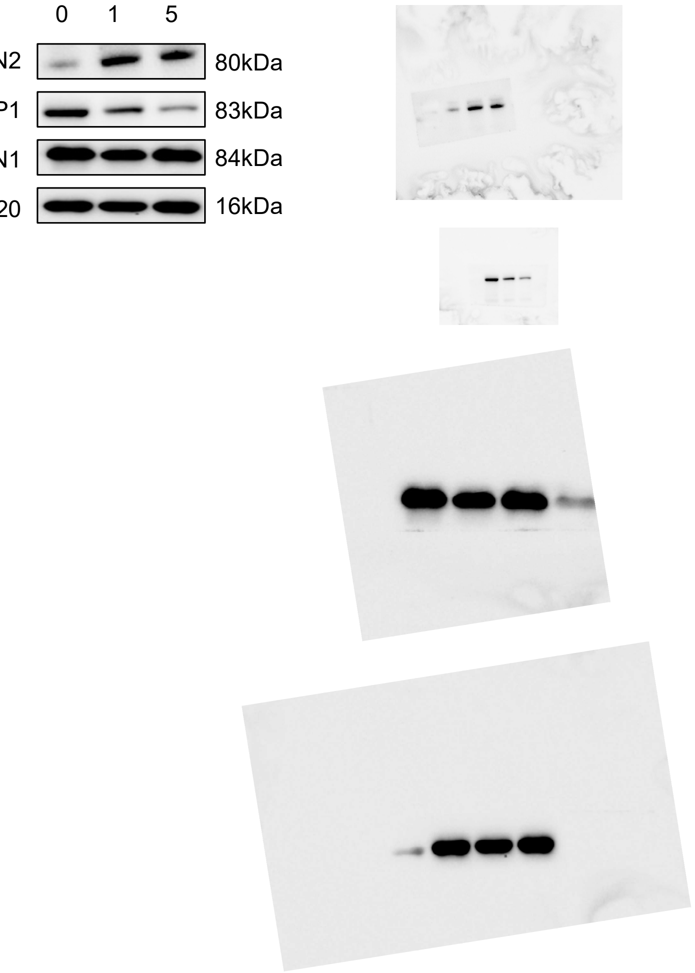

Supplementary Figure 3C

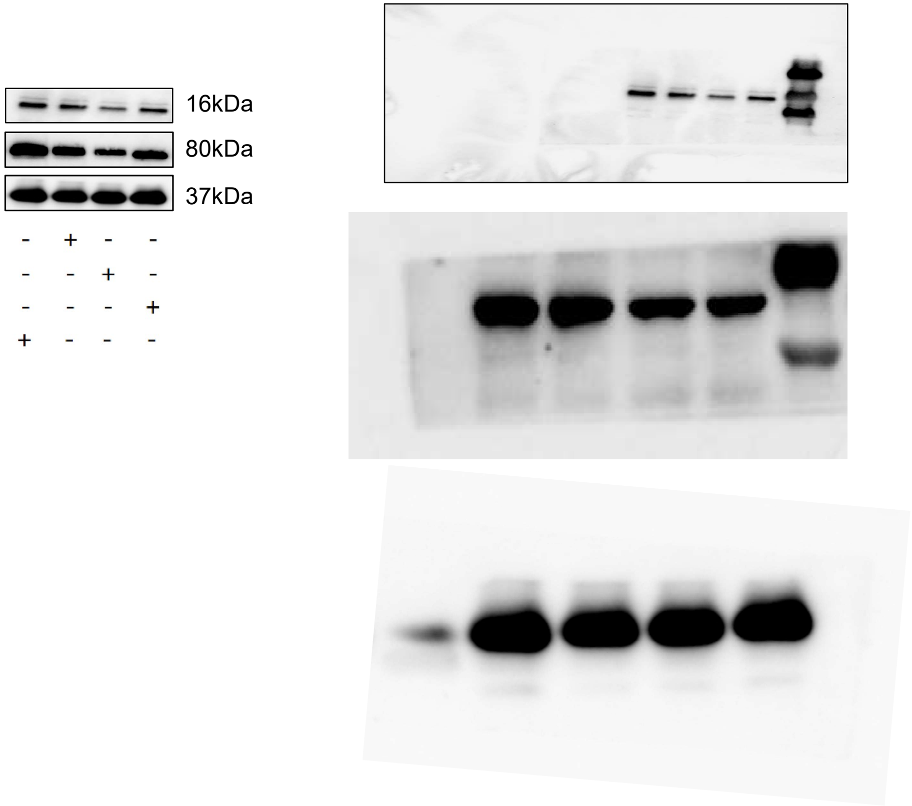

Supplementary Figure 3P

DRP1

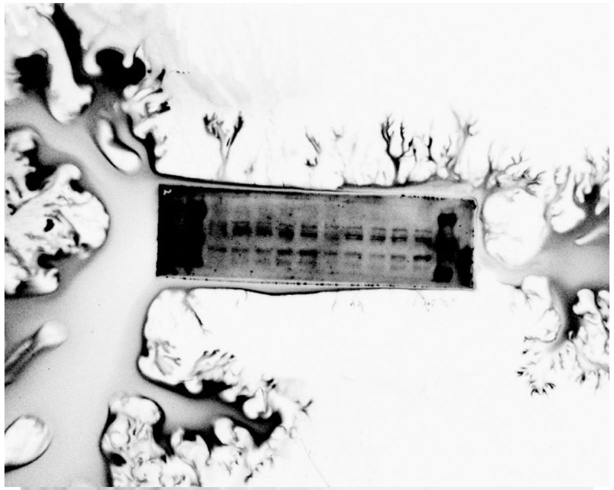

MFN2

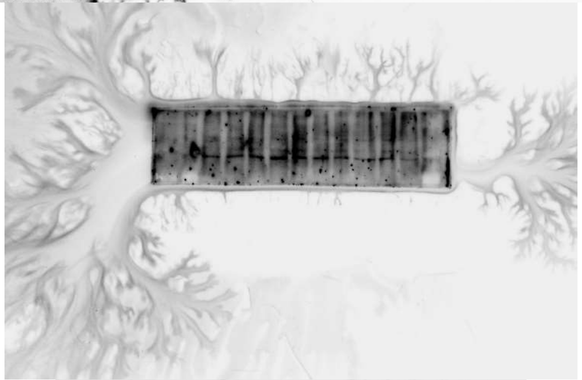

β-actin

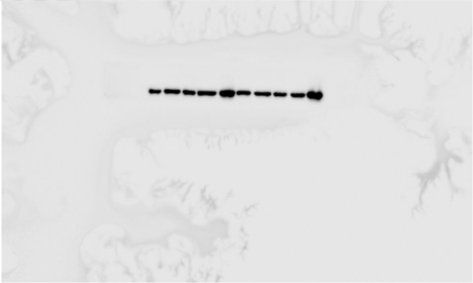

Supplementary 4E

Miro2

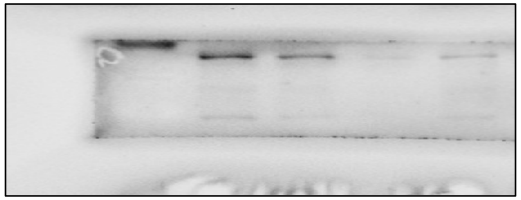

GAPDH

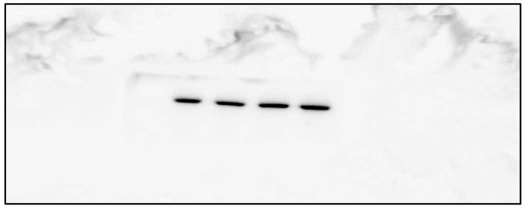

Supplementary 6E

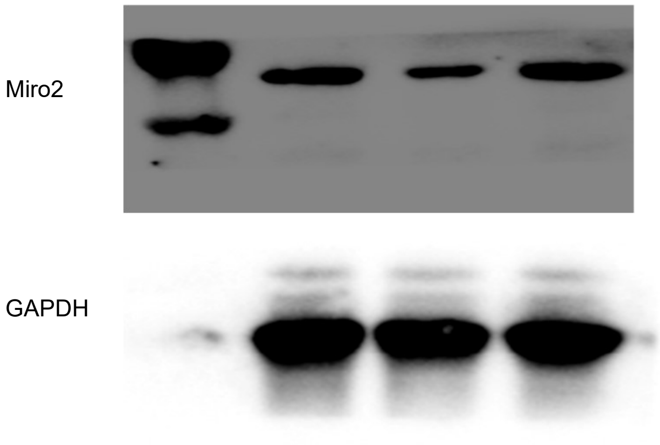

Supplement: Supplementary file 4 — Original WB band [file 41419_2024_7167_MOESM4_ESM.pdf]
